# Recent Advances in Engineering of 2D Materials‐Based Heterostructures for Electrochemical Energy Conversion

**DOI:** 10.1002/advs.202302301

**Published:** 2023-09-24

**Authors:** Yujia Zhang, Kunkun Nie, Lixin Yi, Binjie Li, Yanling Yuan, Zhengqing Liu, Wei Huang

**Affiliations:** ^1^ Frontiers Science Center for Flexible Electronics Xi'an Institute of Flexible Electronics (IFE) Northwestern Polytechnical University Xi'an 710129 China

**Keywords:** electrochemical energy conversion, heterointerface, heterostructure, synergistic effect, 2D material

## Abstract

2D materials, such as graphene, transition metal dichalcogenides, black phosphorus, layered double hydroxides, and MXene, have exhibited broad application prospects in electrochemical energy conversion due to their unique structures and electronic properties. Recently, the engineering of heterostructures based on 2D materials, including 2D/0D, 2D/1D, 2D/2D, and 2D/3D, has shown the potential to produce synergistic and heterointerface effects, overcoming the inherent restrictions of 2D materials and thus elevating the electrocatalytic performance to the next level. In this review, recent studies are systematically summarized on heterostructures based on 2D materials for advanced electrochemical energy conversion, including water splitting, CO_2_ reduction reaction, N_2_ reduction reaction, etc. Additionally, preparation methods are introduced and novel properties of various types of heterostructures based on 2D materials are discussed. Furthermore, the reaction principles and intrinsic mechanisms behind the excellent performance of these heterostructures are evaluated. Finally, insights are provided into the challenges and perspectives regarding the future engineering of heterostructures based on 2D materials for further advancements in electrochemical energy conversion.

## Graphical abstract

2D materials‐based heterostructures, including 2D/0D, 2D/1D, 2D/2D, and 2D/3D, can produce the synergistic effect and heterointerface effect, and overcome the instinct restriction of individual 2D materials, thus can serve as potential electrocatalysts in the field of energy conversion, such as hydrogen evolution reaction, oxygen evolution reaction, carbon dioxide reduction reaction, nitrogen reduction reaction, etc.

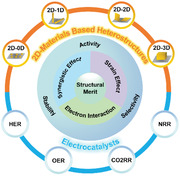

## 1 Introduction

**Figure 1:**
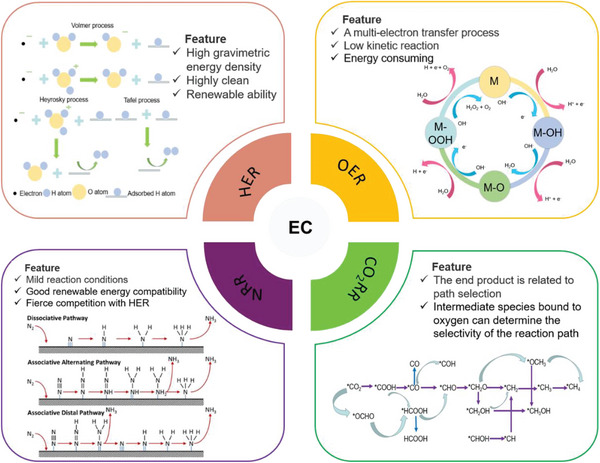
Schematic diagram of energy conversion including four typical kinds of reaction principles. CO_2_RR: Reproduced with permission.^[^ ^5^ ^]^ Copyright 2021, American Chemical Society. NRR: Reproduced with permission.^[^ ^6^ ^]^ Copyright 2016, Elsevier B.V.

Nowadays, emerging environmental issues, such as climate change and the NO*_x_*/CO_2_ pollution caused by the massive consumption of fossil energy, have become inescapable for human beings.^[^ ^1^ ^]^ The rising awareness and social responsibility regarding environmental protection have prompted countries to reach a consensus on sustainable development.^[^ ^2^ ^]^ To address the strong demand for fossil energy, numerous studies have focused on electrochemical energy conversion over the past few decades. These studies encompass various areas including hydrogen evolution reaction (HER), oxygen evolution reaction (OER), overall water splitting (OWS), carbon dioxide reduction reaction (CO_2_RR), and nitrogen reduction reaction (NRR), among others.^[^ ^3^, ^4^ ^]^ **Figure** **1** shows the typical four representative reaction principles. Although energy conversion theories are generally considered ideal, it is important to acknowledge the existing shortcomings such as low conversion efficiency and high investment costs, which cannot be overlooked. Therefore, electrocatalysts play a key role in the application and development of electrochemical energy conversion, as the efficiency of electrochemical energy conversion heavily depends on their performance.

**Figure 2:**
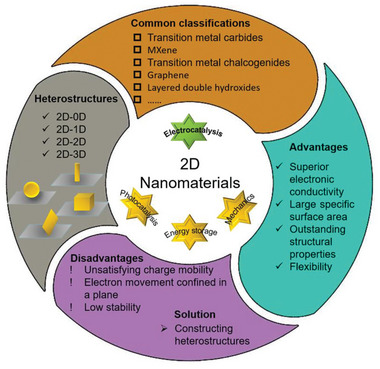
Common classification, advantages, disadvantages, and solutions of 2D materials, and construction modes of heterostructures based on 2D materials.

2D materials are composed of one or several atomic/molecular layers connected by strong covalent or ionic bonds within the layers, and they are stacked by van der Waals (vdW) forces between the layers.^[^ ^7^ ^]^ With the advancement of sustainable energy, the exploration of 2D materials in electrochemical energy conversion has garnered extensive attention due to their unique structure and electronic properties. 2D materials, such as phosphorene,^[^ ^8^, ^9^ ^]^ hexagonal boron nitride (h‐BN),^[^ ^10^ ^]^ transition metal dichalcogenides (TMDs),^[^ ^11^, ^12^ ^]^ layered metal oxides,^[^ ^13^ ^]^ 2D metal–organic framework (MOF),^[^ ^14^, ^15^ ^]^ and 2D covalent organic framework (COF)^[^ ^16^, ^17^ ^]^ have emerged in recent years, demonstrating significant superiority in electrochemical energy conversion (**Figure** **2**). Compared to bulk or other dimensional materials, 2D materials have, among others, the following advantages for electrochemical energy conversion: i) A larger lateral dimension enables 2D materials to possess a higher specific surface area, leading to a much higher utilization rate of atoms. This is conducive to exposing more active sites and improving catalytic efficiency;^[^ ^18^ ^]^ ii) Excellent mechanical properties can help maintain the durability of catalysis;^[^ ^19^ ^]^ iii) High conductivity can improve the electrocatalytic reaction rate;^[^ ^20^ ^]^ iv) The atomic‐level thickness is conducive to an in‐depth study of the reaction process.^[^ ^21^ ^]^ Based on the unique properties mentioned above, researchers are diving into exploring the functions of 2D materials to develop novel electrocatalysts with improved electrocatalytic activity and stability.

Although most of the 2D materials related to electrocatalysis exhibit ultrahigh catalytic behavior, very few 2D nanocatalyst products can maintain long‐term stable and high catalytic activity in reality due to inherent limitations. These limitations include structural stability, electron transfer rate, and anisotropy (Figure 2). Regarding structural stability, 2D materials may undergo chemical or structural changes during their application, which can lead to a reduction of catalytic activity. For example, Liu et al. reported that after the HER reaction of WS_2_ under acidic conditions, oxidation products such as WO*_x_* and SO_4_^2−^ formed on the catalyst surface as observed through XPS (X‐ray photoelectron spectroscopy). These oxidation products weaken the activity and affect catalytic performance.^[^ ^22^ ^]^ As for the electron transfer rate, it has been found that the reaction rate constant at the edges of TMDs is much higher than that of the basal plane, resulting in higher activity of the edge atoms compared to the base plane atoms. Furthermore, previous reports indicate that engineering the phase of TMDs, such as 1T and 1T′, can improve the electron transfer rate.^[^ ^23^, ^24^ ^]^ Regarding anisotropy, the catalytic performance of 2D materials is manifested differently on the active edge surface compared to the inert base surface. Therefore, obtaining a high proportion of exposed edge active sites or activating the active sites on the basal surface to improve their catalytic performance has become one of the research hotspots in the field of 2D materials. Additionally, due to the high reduction potential of graphene, g‐C_3_N_4_, and MXene, it is difficult for them to undergo redox reactions in the potential interval of oxygen reduction reaction (ORR), HER, OER, and CO_2_RR. To address the disadvantages of individual 2D materials, there has been increasing emphasis on constructing heterostructures based on 2D materials by combining them with other 0D, 1D, 2D, or 3D components.^[^ ^25^ ^]^

A heterostructure refers to a combination of different materials, wherein at least two distinct materials are physically or chemically bonded together to form a junction interface.^[^ ^26^ ^]^ The integration of materials in heterostructures introduces a variety of novel characteristics that are absent in individual components. These characteristics include but are not limited to, the following: i) Tailored electronic properties: When two or more materials are combined into a heterostructure, the electronic properties can be extensively tailored.^[^ ^27^ ^]^ For example, a prepared heterostructure can create a structure with a bandgap that differs from that of the individual components. Furthermore, electronic structures at the contact can be positively altered, resulting in improved activity and selectivity of catalytic reactions. These qualities make heterostructured electrocatalysts more effective in the field of energy conversion. ii) Improved strength and durability: Heterostructures also exhibit superior mechanical strength as they distribute stresses between the individual components.^[^ ^28^ ^]^ Consequently, the stability and durability of the heterostructure can be increased accordingly. iii) Control over interfaces: Heterostructures provide control over the structure and properties at the interfaces between components.^[^ ^29^ ^]^ Precisely tuning interactions between different materials influences the electronic properties of the structure. Overall, heterostructures offer a wide array of novel features and properties that are absent in individual components, making them valuable for various electrocatalysis applications, such as HER, OER, OWS, CO_2_RR, NRR, and others. Recent electrochemical energy conversion applications using heterostructures based on 2D materials are summarized in **Table** **1**. It can be observed that the electrocatalytic performance of heterostructures based on 2D materials, constructed with optimized components, is significantly improved compared to that of individual 2D materials.

**Table 1:** Summary of recent studies on 2D materials‐based heterostructures for electrochemical energy conversion.

Heterostructures	Type of junction	Synthesis methods	Structural features	Unique properties	Applications	Performance	Refs.
MoS_2_/rGO	2D–0D	Solvothermal reaction	Small and uniform MoS_2_ distributed on the rGO sheet	Synergistic effect	HER	*η*_10_ = 0.1 V, TS = 41 mV dec^−1^	[35]
rGO/1T′‐MoS_2_/CeO_2_	2D–0D	Wet‐chemical method	CeO_2_ NPs combined with 1T′‐MoS_2_ monolayers on rGO	Proper energy band structure, synergistic effect	HER	Onset potential = −89 mV, TS = 43 mV dec^−1^	[24]
MoS_2_/Mo_2_C	2D–0D	Exfoliation method and a thermal treatment	2D MoS_2_ flakes modified by Mo_2_C NPs	Improved mechanical robustness and the good electrical contact	HER	*η*_1000_ = 412 mV, TS = 60 mV dec^−1^	[36]
Pt/MoS_2_	2D–0D	One‐pot chemical method	Mo atoms are exactly displaced by single Pt atoms	Refine the adsorption behavior of H atoms	HER	*η*_10_ = 0.14 V, TS = 96 mV dec^−1^	[37]
CoS_2_/CuS	2D–0D	Hydrothermal‐electro‐deposition	CuS nanosheets decorated with CoS_2_ nanoparticles	Regulatory effect of heterostructure on electrons and energy bonds	HER	*η*_10_ = 62 mV, TS = 46 mV dec^−1^	[38]
Au@MoS_2_	2D–1D	Colloidal synthesis approach	MoS_2_ monolayers formed on the surface of Au nanorods	Efficient electron transfer	HER	*η*_10_ = 178 mV, TS = 43.3 mV dec^−1^	[39]
MoS_2_/CNTs	2D–1D	Liquid‐phase synthesis approach	MoS_2_ nanosheets attached to the surface of CNTs	High electron mobility	HER	*η*_10_ = 79 mV, TS = 33.5 mV dec^−1^	[40]
Co/NCNT/g‐C_3_N_4_	2D–1D	Sol–gel process	Co NPs encapsulated with carbon nanotubes/graphene	High surface area and large pore volume	HER ORR	HER: *η*_10_ = 123 mV, TS = 65 mV dec^−1^ ORR: half‐wave potential = 0.85 V	[41]
MoSe_2_/r‐GO/CNT	2D–1D	Spray pyrolysis process	Ternary heterostructures and porous structure	Higher catalytic efficiency than binary materials	HER	*η*_10_ = 0.24 V, TS = 53 mV dec^−1^	[42]
CoP/NSGO	2D–1D	Thermal decomposition method	CoP nanorods in situ grown on N, S co‐doped graphene oxide	Synergistic effect	HER	*η*_10_ = 45 mV, TS = 52 mV dec^−1^	[43]
Ni_2_P@CoP	2D–1D	Hydrothermal reaction, chemical bath deposition, and phosphorization	Ni_2_P nanosheets@CoP nanowires supported on carbon cloth substrates	Electronic interaction, synergistic effect	HER	*η*_10_ = 55 mV, TS = 48 mV dec^−1^	[44]
MoS_2_/Ni–Co LDH	2D–2D	Two‐step hydrothermal method	Heterostructure plane is aligned perpendicular to the base material	Water molecules can be decomposed faster	HER	*η*_100_ = 78 mV, TS = 76.6 mV dec^−1^	[45]
MoS_2_/rGO	2D–2D	Solvothermal method	MoS_2_ uniformly covered the surface of rGO	Abundant active catalytic edges	HER	*η*_10_ = −0.19 V, TS = 95 mV dec^−1^	[46]
MoP/MoS_2_	2D–2D	Hydrothermal method	Formed interface between MoP and MoS_2_	Δ*G*_H_ ^a)^ comes near zero	HER	Neutral: *η*_10_ = 96 mV, TS = 48 mV dec^−1^ Alkaline: *η*_10_ = 54 mV, TS = 58 mV dec^−1^ Acid: *η*_10_ = 69 mV, TS = 61 mV dec^−1^	[47]
MoS_2_/MoSe_2_	2D–2D	Electron beam evaporation	Vertical growth heterostructures	High electrical conductivity	HER	TS = 75 mV dec^−1^, TOF_0V_ = 0.013 s^−1^	[48]
Co–N–Ni_9_S_8_/Nb_2_O_5_	2D–2D	Two‐step hydrothermal method	Doping materials with N atoms	Regulate local charge distribution and electronic properties	HER	*η*_10_ = 171 mV, TS = 69 mV dec^−1^	[49]
Cu–N–Ni_9_S_8_/Nb_2_O_5_	2D–2D	Two‐step hydrothermal method	Doping materials with N atoms	Modulate local charge distribution and electronic properties	HER	*η*_10_ = 109 mV, TS = 51 mV dec^−1^	[49]
CoP/Co–MOF	2D–2D	Electrodeposition and hydrothermal treatment	Suitable interfacial sites at the specific synthesis condition	Wide range of pH and satisfactory durability	HER	Neutral: *η*_10_ = 106 mV Alkaline: *η*_10_ = 206 mV Acid: *η*_10_ = 52 mV	[50]
Nitrogen‐doped MoS_2_/graphene	2D–2D	Template method	N‐MoS_2_ nanosheets merged with graphene	Trifunctional electrocatalytic performance	HER OER ORR	HER *η*_10_ = 243 mV, TS = 82.5 mV dec^−1^ OER *η*_10_ = 161 mV ORR *η*_10_ = 177 mV	[51]
Mo_2_C/graphene	2D–2D	Electrochemical vapor deposition	Mo grows vertically on the surface of the graphene	Strong electronic coupling of graphene and Mo_2_C	HER	*η*_10_ = 236 mV, TS = 73 mV dec^−1^	[52]
CuS/graphdiyne	2D–2D	Hydrothermal method and in situ polymerization	Graphdiyne‐coated electrocatalyst	Tight interaction	HER	*η*_10_ = 106 mV, TS = 63.8 mV dec^−1^	[53]
C_3_N_4_/N doped graphene	2D–2D	Vacuum filtration method	3D film via combining porous C_3_N_4_ layers with nitrogen‐doped graphene nanosheet	Synergistic effect, rich active sites, porous structure, and 3D conductive network	HER	*η*_10_ = 80 mV, TS = 49.1 mV dec^−1^	[54]
Ni–Mo–S/C	2D–3D	Hydrothermal method	Nanostructured Ni‐Mo‐S grown on 3D conductive carbon fiber cloth substrate	Remarkable electrocatalytic activity and stability	HER	*η*_10_ = 200 mV, TS = 85.3 mV	[55]
CoP/CoMoP	2D–3D	Dissolution‐regrowth and phosphorization	Nanocages structure	HER over a broad PH range	HER	Neutral: *η*_10_ = 151 mV, TS = 74.5 mV dec^−1^ Alkaline: *η*_10_ = 72 mV, TS = 60.3 mV dec^−1^ Acid: *η*_10_ = 44 mV, TS = 51.2 mV dec^−1^	[56]
MoS_2_/rGO	2D–3D	One‐pot hydrothermal method	Hierarchical MoS_2_ nanosheets on 3D reduced graphene oxide aerogel structure	Large surface areas and many active sites	HER	*η*_10_ = 286 mV, TS = 77 mV dec^−1^	[57]
MoS_2_/nanoporous gold	2D–3D	CVD method	Porous alloy and uneven structure	Low‐resistance Ohmic contact	HER	Onset = −118 mV, TS = 46 mV dec^−1^	[58]
MoS_2_/Mo_2_C	2D–3D	Hydrothermal method and CVD	MoS_2_ microspheres made of MoS_2_ NSs and decorated by Mo_2_C NPs	Spherical morphology is beneficial for the access of reactants and the release of H_2_	HER	Alkaline: *η*_1000_ = 220 mV TS = 44 mV dec^−1^ Acid: *η*_1000_ = 227 mV TS = 53 mV dec^−1^	[59]
Mo–W–P/Carbon cloth	2D–3D	Hydrothermal method	3D hierarchical and porous structure	Strong synergistic effect, 3D conductive scaffolds	HER	*η*_100_ = 138 mV, TS = 52 mV dec^−1^	[60]
MoS_2_/Co_3_O_4_	2D–3D	Calcination, hydrothermal method	3D Co_3_O_4_ decorated with MoS_2_ nanosheets	Strong electrostatic interaction, short diffusion pathway	HER	HER: *η*_20_ = 205 mV, TS = 98 mV dec^−1^	[61]
Crystalline/amorphous hollow MoS_2_	2D–3D	Solvothermal method	Crystalline/amorphous MoS_2_ nanosheets self‐assembling into 3D hollow structure	Abundant active sites	HER	Onset potential = −112 mV, TS = 45 mV dec^−1^	[11]
Ni_3_N/AG‐BP	2D–0D	Ball‐milling	Ni_3_N particles supported by activated graphene–black phosphorus nanosheets	Controlled direct bandgap, high electron mobility, the formation of NiOOH layers in the OER process	OER	*η*_10_ = 233 mV, TS = 42 mV dec^−1^	[62]
NiS/Bi_2_WO_6_	2D–0D	Hydrothermal and calcined synthesis	NiS nanoparticles tightly anchoring Bi_2_WO_6_ nanosheets	Strong coupling, more exposed active sites, high charge transfer rates, and good stability	OER	*η*_10_ = 527 mV, TS = 238 mV dec^−1^	[63]
Ni_2_P/NiFeP/NF	2D–0D	Hydrolysis, in situ growth, and phosphorization	Ni_2_P nanoparticles inserted NiFeP nanosheets on Ni foam	Rich interfaces, high pore volumes, specific surface areas, fast mass, and electron transfers	OER	*η*_50_ = 250 mV, TS = 35 mV dec^−1^	[64]
PQDs/MoS_2_	2D–0D	One‐step electrochemical approach	Phosphorene quantum dots interspersed with MoS_2_ nanosheets	Unique morphology benefitting charge transfer	OER	*η*_10_ = 1.6 V, TS = 46 mV dec^−1^	[65]
CoO*_x_* nanotube/nanosheet	2D–1D	Solution‐based 1D crystal growth method, in situ etching	Ultrathin CoO*_x_* nanosheets assembled into a nanotube structure	Bio‐inspired architecture facilitates a highly active Co^2+^ electronic structure	OER	*η*_51.2_ = 1.65 V, TS = 75 mV dec^−1^	[66]
CeO_2_/Co_3_O_4_	2D–1D	Etching, thermal treatment	2D nanosheets self‐assembled into 1D nanotubes to form a hierarchical heterostructure	Strongly coupled p–n heterointerfaces, fast electron transfer	OER	*η*_10_ = 265 mV, TS = 68.1 mV dec^−1^	[67]
NiCo/Ni/CuO/CF	2D–1D	Calcination, electro‐deposition, and hydrothermal method	Ni‐Co hydroxide nanosheets on Ni coated by CuO nanowires grown on Cu foam	Large surface area, more exposure of active sites, and high electrical conductivity	OER	*η*_10_ = 240 mV, TS = 37.9 mV dec^−1^	[68]
NiFeO*_x_* LDH/CuO/CF	2D–1D	Solution‐based method	NiFeO*_x_* nanosheets codeposited onto the CuO nanowires at a planar copper foil	High specific area, good electrical contact between the electrocatalyst and the conductive substrate, good stability	OER	*η*_100_ = 300 mV, TS = 36 mV dec^−1^	[69]
NiMn LDH/NiCo_2_O_4_	2D–1D	Hydrothermal‐annealing‐hydrothermal	NiMn LDH nanosheets grown on NiCo_2_O_4_ nanowires	High valence, synergistic effect, high electrical conductivity, and efficient active sites	OER	*η*_10_ = 310 mV, TS = 99 mV dec^−1^	[70]
CoMnFe nanosheet‐nanowire	2D–1D	Hydrothermal method	CoMnFe layered triple hydroxide with nanosheet‐nanowire structure grown on Ni foam	Uniform active sites distribution, high electronic conductivity, and open channels for gas releasing	OER	*η*_100_ = 226 mV, TS = 55 mV dec^−1^	[71]
Co_4_N/CoO_2_	2D–1D	Nitridation method	Metallic Co_4_N nanowires inside with a thin cobalt oxides/hydroxides shell	Porous structure, fast charge transport	OER	*η*_10_ = 257 mV, TS = 44 mV dec^−1^	[72]
FeNi LDH/GO	2D–2D	Hydrothermal method, anion exchange processes	GO nanosheets assembled with the FeNi double hydroxide layers	Charge balancing interlayers, electrostatic attraction, synergistic interactions	OER	*η*_10_ = 0.21 V, TS = 40 mV dec^−1^, TOF_0.3 V_ = 0.98 s^−1^	[73]
NiO@NiFe LDH	2D–2D	Two‐step hydrothermal method and calcination	Core–shell arrays on NF	Synergistic interactions, 3D porous structure	OER	*η*_10_ = 265 mV, TS = 72 mV dec^−1^	[74]
Ni–BDC/Ni(OH)_2_	2D–2D	Sonication‐assisted solution process	Ni‐BDC nanosheets attached to the flat Ni(OH)_2_ nanosheets	Strong electron interactions, modified electronic structure, and further oxidation states	OER	*η*_10_ = 320 mV, TS = 41 mV dec^−1^	[75]
FeCo LDH/CoO	2D–2D	Low‐temperature phosphatization reaction	2D FeCo layered double hydroxide, cobalt oxide nanosheets	Rational component design, in‐plane component tunability, the strong interfacial coupling effect	OER	*η*_10_ = 219 mV, TS = 52 mV dec^−1^	[76]
Co_1.8_Ni(OH)_5.6_@Co_1.8_NiS_0.4_(OH)_4.8_	2D–2D	Ethanol‐modified surface sulfurization method	Core–shell structure, ultrathin CoNi hydroxysulfide shell fabricated on CoNi hydroxide nanosheets	High activities, rapid kinetics, remarkable durability	OER	*η*_10_ = 274 mV, TS = 45 mV dec^−1^	[77]
MoS_2_/g‐C_3_N_4_	2D–2D	Density functional theory calculations	Layered g‐C_3_N_4_ coupled on the vertical direction of MoS_2_ monolayers	Strong electronic coupling, improved electronic properties	OER	*η*_10_ = 0.78 V	[78]
Ni_2_P@C/G	2D–3D	Solvothermal treatment, calcination, and phosphorization	Ni_2_P nanocrystals supported on graphene	Enlarged active surface area, increased exposure to active sites, an accelerated charge transport	OER	*η*_10_ = 285 mV, TS = 44 mVdec^−1^	[79]
CoS_2_@MoS_2_	2D–3D	Solvothermal and hydrothermal methods	MoS_2_ nanosheets vertically grown on the surface of CoS_2_ to form the hollow nanocubes	Large effective surface area and long‐term stability	OER	*η*_10_ = 347 mV, TS = 147 mV dec^−1^	[80]
CoNiFeO*_x_*–NC	2D–3D	Ion‐exchange based method	3D octahedral MOF crystals encapsulated by 2D ternary MOF shell layer	Rich active sites, the refined binding strength of O species, low energy barriers	OER	*η*_10_ = 350 mV, TS = 80 mV dec^−1^	[81]
Ag/Co(OH)_2_@NC/CC	2D–3D	In situ growth, pyrolysis, acid leaching, in situ self‐transformation, and redox reduction	Co(OH)_2_ nanosheets decorated with Ag nanoparticles supported on nitrogen‐doped carbon nanoflake arrays on carbon cloth	Large surface area massive ion transport pathways, and the high electrical conductivity	OER	*η*_50_ = 300 mV, TS = 79.8 mV dec^−1^	[82]
NiFe LDH nanocages	2D–3D	One‐pot self‐templated method	NiFe LDH double‐shelled nanocages assembled by ultrathin nanosheets	Hierarchical hollow structures, double‐shelled structure, massive ultrathin nanosheets, reduced charge transfer resistance	OER	*η*_50_ = 265 mV, TS = 64.1 mV dec^−1^	[83]
N‐doped carbon/Co‐decorated N‐doped graphitic carbon	2D–3D	Epitaxial growth, carbonization, acidic leaching	Double‐shelled nanocages	High activity, rapid diffusion kinetics	OER ORR	OER: *η*_10_ = 1.64 V, TS = 91 mV dec^−1^ ORR: E_1/2_ = 0.82 V, TS = 51 mV dec^−1^	[84]
Co_2_P_2_O_7_@N, P co‐doped carbon	2D–3D	Ligands exchange reaction	Mott–Schottky nanocages heterostructures	High intrinsic activity, improved charge polarization	OER	*η*_50_ = 310 mV, TS = 49.1 mV dec^−1^, TOF_320Mv_ = 0.103 s^−1^	[85]
Ag@CoMo LDH	2D–3D	Template method and spontaneous strategy	Metal‐support nanocages heterostructure consisting of Ag and CoMo‐LDH	Rapid transfer of the electron and mass, high activity of sites, more appearance of new active sites, and the stable heterointerfaces	OER	*η*_10_ = 205 mV, TS = 45.7 mV dec^−1^	[86]
Ni_3_FeN/rGO	2D–0D	One‐step nitrogenization	Ni_3_FeN nanoparticles on reduced graphene oxide nanosheets	Substantial active sites, effective mass transfer, high electrical conductivity	OWS	HER: *η*_10_ = 94 mV, TS = 90 mV dec^−1^ OER: *η*_10_ = 270 mV, TS = 54 mV dec^−1^ OWS: *η*_10_ = 1.60 V	[87]
CoP_2_/rGO	2D–0D	Low‐temperature phosphatization reaction	CoP_2_ NPs grown on reduced graphene oxide sheets	Favorable reaction kinetics, good ability, and stability	OWS	HER *η*_10_ = 97 mV, TS = 50 mV dec^−1^ OER *η*_10_ = 1.55 V, TS = 96 mV dec^−1^ OWS *η*_10_ = 1.56 V,	[88]
Ni/N‐doped graphene	2D–0D	High‐temperature annealing treatment	Ni NPs encapsulated in few‐layer nitrogen‐doped graphene	Synergistic effects, high activity, and good durability	OWS	HER: *η*_10_ = 250 mV, TS = 160 mV dec^−1^ OER: *η*_10_ = 1.52 V, TS = 45 mV dec^−1^ OWS: *η*_10_ = 1.6 V	[89]
NiP/NSG	2D–0D	Solution‐phase method, ex situ sonication	Ni_2_P nanocrystals encapsulated in N and S co‐doped graphene (NSG) nanosheets	Interaction and synergistic effect, large specific surface area, high catalytic activity	OWS	HER: *η*_20_ = 110 mV, TS = 43 mV dec^−1^ OER: *η*_10_ = 240 mV, TS = 47 mV dec^−1^ OWS: *η*_10_ = 1.572 V	[90]
NiFe_2_O_2_/NiFe LDH	2D–0D	One‐step solvothermal approach	NiFe_2_O_2_ NPs attached to NiFe LDH nanosheets	Strong coupling effects, rich active sites, high catalytic reactivity, good durability	OWS	HER: *η*_10_ = 101 mV, TS = 67.1 mV dec^−1^ OER: *η*_100_ = 213 mV, TS = 28.2 mV dec^−1^ OWS: *η*_10_ = 1.535 V	[91]
MoS_2_/Ni_3_S_2_	2D–0D	One‐pot solvothermal reaction	MoS_2_ nanosheets decorated on Ni_3_S_2_ nanoparticles	Rich interfaces	OWS	HER: *η*_10_ = 110 mV, TS = 83.1 mV dec^−1^ OER: *η*_10_ = 218 mV, TS = 88 mV dec^−1^ OWS: *η*_10_ = 1.56 V	[92]
CoNC@MoS_2_/CNF	2D–1D	Carbonization, solvothermal treatment	Thin MoS_2_ nanosheets grafted Co–N–C flakes and grown on electrospun carbon nanofibers	Hierarchical structure, favorable flexibility, high electrical conductivity, synergistic effect, excellent catalytic activities, and good stability	OWS	HER: *η*_10_ = 143 mV, TS = 68 mV dec^−1^ OER: *η*_10_ = 350 mV, TS = 51.9 mV dec^−1^ OWS: *η*_10_ = 1.62 V	[93]
NiCo_2_S_4_@MnO_2_	2D–1D	Hydrothermal reaction	NiCo_2_S_4_ nanowires interspersed in MnO_2_ nanosheets	Rapid electron transfer, excellent stability, improved synergistic effect	OWS	HER: *η*_10_ = 114 mV, TS = 52.5 mV dec^−1^ OER: *η*_100_ = 420 mV, TS = 30.8 mV dec^−1^ OWS: *η*_10_ = 1.504 V	[94]
MoS_2_–Ni_3_S_2_/NF	2D–1D	One‐pot synthesis	Ni_3_S_2_ nanorods integrated with MoS_2_ nanosheets	High exposure of active heterointerfaces, fast charge transport, improved kinetics and activity	OWS	HER: *η*_10_ = 98 mV, TS = 61 mV dec^−1^ OER: *η*_10_ = 249 mV, TS = 57 mV dec^−1^ OWS: *η*_10_ = 1.50 V	[95]
Cu_2_S/Co_9_S_8_	2D–1D	Impregnation, electrodeposition	Co_9_S_8_ nanowires coupled with Cu_2_S nanorods	Interfacial interaction, strong electron interaction, rapid charge transfer	OWS	HER: *η*_10_ = 165 mV, TS = 80.2 mV dec^−1^ OER: *η*_10_ = 195 mV, TS = 78.8 mV dec^−1^ OWS: *η*_10_ = 1.6 V	[96]
NiFe LDH@NiCoP	2D–1D	Hydrothermal‐phosphating	NiFe LDH nanosheets assembled on NiCoP nanowires	Synergistic effect, strong electronic interaction, good activity, and stability	OWS	HER: *η*_10_ = 120 mV, TS = 88.2 mV dec^−1^ OER: *η*_10_ = 220 mV, TS = 48.6 mV dec^−1^ OWS: *η*_10_ = 1.57 V	[97]
NiAl LDH/MoS_2_	2D–2D	Self‐assembly method	NiAl LDH overlapping MoS_2_ nanosheets	Strong electronic coupling, promoted electrical conductivity, improved charge transfer kinetics, good durability	OWS	HER: *η*_10_ = 0.22 V, TS = 67 mV dec^−1^ OER: *η*_10_ = 0.31 V, TS = 56 mV dec^−1^ OWS: *η*_10_ = 1.49 V	[98]
NiFe LDH/MoS_2_	2D–2D	Self‐assembly of 2D building blocks	NiFe LDH overlapping MoS_2_ nanosheets	Strong electronic coupling, promoted electrical conductivity, improved charge transfer kinetics, good durability	OWS	HER: *η*_10_ = 0.18 V, TS = 82 mV dec^−1^ OER: *η*_10_ = 0.25 V, TS = 45 mV dec^−1^ OWS: *η*_10_ = 1.45 V	[98]
NiFe LDH@DG	2D–2D	Electrostatic flocculation	NiFe nanosheet coupled with defective graphene (DG)	Sufficient defective sites, excellent kinetics, increased density of electron transfer	OWS	HER: *η*_20_ = 115 mV, TS = 110 mV dec^−1^ OER: *η*_10_ = 0.21 V, TS = 52 mV dec^−1^ OWS: *η*_10_ = 1.5 V	[99]
rGO/MoS_2_/Pd	2D–2D	Two‐step solvothermal method	Pd NPs anchored onto reduced graphene oxide‐supported MoS_2_ nanosheets	Large interfacial surface area, enhanced overall conductivity, excellent stability	OWS	HER: *η*_10_ = 86 mV, TS = 35.9 mV dec^−1^ OER: *η*_10_ = 245 mV, TS = 42 mV dec^−1^	[100]
MoSe_2_/Ti_3_C_2_	2D–2D	One‐step hydrothermal method	1T MoSe_2_ nanosheets grown on 2H Mxene nanosheets	Plenty of active sites and promoted charge transfer ability	OWS	HER: *η*_10_ = 95 mV, TS = 91 mV dec^−1^ OER: *η*_10_ = 340 mV, TS = 90 mV dec^−1^ OWS: *η*_10_ = 1.64 V	[101]
NiS/G	2D–2D	Pyrolysis, sulfidation process	NiS nanosheets coupled with graphene	Strong interface coupling, massive active sites, promoted electrical conductivity	OWS	HER: *η*_10_ = 70 mV, TS = 82 mV dec^−1^ OER: *η*_10_ = 300 mV, TS = 50.1 mV dec^−1^ OWS: *η*_10_ = 1.54 V	[102]
Mo–NiCo_2_O_4_/Co_5.47_N/NF	2D–2D	Hydrothermal reaction, thermal process	Mo‐doped porous NiCo_2_O_4_/Co_5.47_N nanosheets coated on nickel foam	Reduced chemisorption‐free energy, highly exposed active sites	OWS	HER: *η*_50_ = 170 mV OER: *η*_50_ = 310 mV, TS = 55.1 mV dec^−1^ OWS: *η*_10_ = 1.56 V	[103]
NiFe/NiCo_2_O_4_/NF	2D–2D	Hydrothermal deposition, thermal annealing, electrodeposition	Three‐level hierarchy including NiFe nanosheets, NiCo_2_O_4_ nanoflakes, nickel foams	Rich active sites, enhanced mass transport, rapid dissipation of gases	OWS	HER: *η*_10_ = 105 mV, TS = 88 mV dec^−1^ OER: *η*_1200_ = 340 mV, TS = 38.8 mV dec^−1^ OWS: *η*_10_ = 1.67 V	[104]
MoNi_4_/NF	2D–3D	Hydrothermal method, annealing	Porous MoNi_4_ networks grown on nickel foam	Increased active sites, excellent activity, and stability	OWS	HER: *η*_10_ = 28 mV, TS = 36 mV dec^−1^ OER: *η*_10_ = 1.51 V, TS = 79 mV dec^−1^ OWS: *η*_10_ = 1.58 V	[105]
CoMnCH/NF	2D–3D	Hydrothermal method	Co–Mn carbonate hydroxide nanosheets were grown onto 3D conductive nickel foam	Large active surface area, rich accessible active sites, optimized electronic structure, enhanced activity	OWS	HER: *η*_10_ = 180 mV, OER: *η*_30_ = 294 mV OWS: *η*_10_ = 1.68 V	[106]
Co@N–CS/N–HCP@CC	2D–3D	Electrodeposition, pseudomorphic replication, pyrolysis method	Ultrafine Co nanoparticles embedded within 2D N‐doped carbon nanosheets and 3D N‐doped hollow carbon polyhedra	Massive active sites, synergistic effect	OWS	HER: *η*_10_ = 66 mV, TS = 65 mV dec^−1^ OER: *η*_10_ = 248 mV, TS = 68 mV dec^−1^ OWS: *η*_10_ = 1.545 V	[107]
Co/Co_9_S_8_@S,N‐doped graphene	2D–3D	Pyrolysis method	Co/Co_9_S_8_ core–shell structures anchored on S, N ‐doped graphene nanosheets	High surface area, large pore volume	OWS	HER: *η*_20_ = 350 mV, TS = 82.7 mV dec^−1^ OER: *η*_10_ = 290 mV, TS = 79.7 mV dec^−1^ OWS: FE = ≈100%	[108]
Co–Ni–S–P/graphene	2D–3D	Sulfidation, phosphatization treatment	3D architectural (Co_1−_ *_x_* Ni*_x_*)(OH)_2_ compounds coupled with graphene	Abundant active sites, appropriate dual tuning of Ni and P, high stability and durability	OWS	HER: *η*_10_ = 117 mV, TS = 85 mV dec^−1^ OER: *η*_10_ = 285 mV, TS = 105 mV dec^−1^ OWS: *η*_10_ = 1.65 V	[109]
Cu@C	2D–0D	Ball‐milling	Cu NPs embedded in carbon substrate	Fast charge transfer, promoted the intermediate formation	CO_2_RR	FE = 78%	[110]
SnO_2_/CuS	2D–0D	One‐pot method	SnO_2_ NPs confined on ultrathin CuS nanosheets	Strong electronic interaction, excellent mass activity	CO_2_RR	FE > 85%	[111]
CuSn/C	2D–1D	Hydrothermal process, followed by thermal treatment	CuSn nanowires deposited on carbon	Enhanced adsorption of CO_2_ intermediate, inhibiting H_2_ production, excellent stability	CO_2_RR	FE = 90.2%	[112]
Cu/CuO	2D–1D	Calcination followed by hydrothermal	CuO shell on Cu nanowires	Particularly strong adsorption of the reaction intermediate^a)^ COOH, fast kinetic CO generation, high partial current density	CO_2_RR	FE = 90%	[113]
g‐C_3_N_4_/MWCNT	2D–1D	Solution reaction	g‐C_3_N_4_ nanosheets attached to the multiwall carbon nanotubes	Rich active site, large surface area, and promoted conductivity	CO_2_RR	FE = 60%	[114]
CuSe/g‐C_3_N_4_	2D–2D	Hydrothermal method	CuSe nanoplates anchored on g‐C_3_N_4_ nanosheets	A large specific area and fast electron transfer	CO_2_RR	FE = 85.28%	[115]
CoS_2_/MoS_2_	2D–0D	Hydrothermal method	CoS_2_ NPs decorated on MoS_2_ nanosheets	Strong interaction, decreased energy barrier, self‐driven charge transfer	NRR	NH_3_ yield = 54.7 µg h^−1^ mg^−1^, FE = 20.8%	[116]
CoS_2_/NS–G	2D–0D	Annealing method	CoS_2_ NPs fabricated on nitrogen‐ and sulfur‐doped reduced graphene	Strong bridging bonds (Co–N/S–C), rapid reaction kinetics	NRR	NH_3_ yield = 25.0 µg h^−1^ mg^−1^, FE = 25.9%	[117]
MoO_2_/rGO	2D–0D	Microwave‐assisted hydrothermal method	MoO_2_ NPs on reduced graphene oxide	Strong electronic interactions with ^a)^N_2_H, reduced energy barrier for ^a)^N_2_H formation	NRR	NH_3_ yield = 37.4 µg h^−1^ mg^−1^, FE = 6.6%	[118]
Cr_2_O_3_/rGO	2D–0D	Hydrothermal followed by annealing	Cr_2_O_3_ NPs on reduced graphene oxide	Good dispersion, rich active sites, enhanced conductivity	NRR	NH_3_ yield = 33.3 µg h^−1^ mg^−1^, FE = 7.33%	[119]
Ni@MXene	2D–0D	Etching, exfoliation, thermal reduction	Ni NPs loaded on V_4_C_3_T*_x_* Mxene	Synergistic effect, the surface O vacancy	NRR	NH_3_ yield = 21.29 µg h^−1^ mg^−1^, FE = 14.86%	[120]
MoS_2_/C_3_N_4_	2D–2D	Liquid exfoliation, hydrothermal method	MoS_2_ nanosheets coupled with C_3_N_4_ nanosheets	Strong coupling interactions, promoted stabilization, decreased reaction energy barrier	NRR	NH_3_ yield = 18.5 µg h^−1^ mg^−1^, FE = 17.8%	[121]
MoS_2_/rGO	2D–2D	Thermal treatment	MoS_2_ nanosheets coupled with reduced graphene oxide	Coupling effects, high surface area, strong electrochemical stability	NRR	NH_3_ yield = 24.82 µg h^−1^ mg^−1^, FE = 4.56%	[122]
1T’‐MoS_2_/Ti_3_C_2_	2D–2D	Etching, hydrothermal method	1T′‐MoS_2_ nanosheets supported by Ti_3_C_2_	Excellent selectivity and stability	NRR	NH_3_ yield = 31.96 µg h^−1^ mg^−1^, FE = 30.75%	[123]
TiO_2_/Ti_3_C_2_T*_x_*	2D–2D	One‐step hydrothermal oxidation process	TiO_2_ nanosheets grown on Ti_3_C_2_T*_x_* nanosheets	Enhanced electron transfer, large surface active area	NRR	NH_3_ yield = 44.17 µg h^−1^ mg^−1^, FE = 44.86%	[124]
FePc/C	2D–3D	Pyrolysis method, low‐temperature annealing	FePc on the 3D carbon substrate	More exposure of active sites	NRR	NH_3_ yield = 10.25 µg h^−1^ mg^−1^, FE = 10.5%	[125]
CoS_2_/MoS_2_	2D–3D	Hydrothermal method	Flower‐like microspheres consisting of vertically interconnected CoS_2_ (with the walnut shape) and MoS_2_ nanosheets	Synergistic effect, abundant active sites, interface electronic coupling,	NRR	NH_3_ yield = 38.61 µg h^−1^ mg^−1^, FE = 34.66%	[126]
NiS@MoS_2_	2D–3D	Annealing, solvothermal method	MoS_2_ nanosheets grown on NiS_2_–NiS hierarchical microspheres	Plenty of active sites, high structural porosity, accessible transport channels, and good stability	NRR	NH_3_ yield = 9.66 µg h^−1^ mg^−1^, FE = 14.8%	[127]

^a)^ TS stands for Tafel slope, FE stands for Faraday efficiency, and η_10_ stands for the overpotential at the current density of 10 mA cm^−2^.

Through comparison and analysis, the structural merits of heterostructures based on 2D materials are reflected in the following three aspects: i) Synergistic effect: In heterostructures, bond cooperation between different components can improve the electron transfer rate. By combining 2D materials with other components, the electrical conductivity, hydrophilicity, chemical stability, and density of active sites in heterostructures can be regulated.^[^ ^30^ ^]^ ii) Strain effect: Different components and crystal structures in heterostructures can cause lattice strains, such as tensile and compressive strains, which affect the adsorption energy of sites for intermediates and enhance the catalytic activity of 2D materials.^[^ ^31^ ^]^ iii) Electron interaction: In heterostructures, different energy band arrangements of various components can lead to charge transfer at interfaces, which is conducive to surface electron modulation in heterostructures based on 2D materials. Therefore, the structural and electronic merits of heterostructures based on 2D materials show great promise in maximizing the exposure of active sites and in optimizing the energy and kinetics of electrocatalytic reactions.

In this review, we summarize the functional principles and characteristics of heterostructures based on 2D materials for the applications of electrochemical energy conversion. Meanwhile, we provide relevant and recent examples to illustrate these principles. We emphasize the various roles that heterostructures based on 2D materials play in reaction processes and how they can significantly impact catalytic efficiency. Moreover, we highlight the synthesis approaches of heterostructures based on 2D materials. In recent years, several studies have documented the advancements in the utilization of 2D materials for energy conversion. For example, Zhang et al. mainly reviewed the latest research on photocatalysis and electrocatalysis of NRR, utilizing various individual 2D material catalysts.^[^ ^32^ ^]^ Similarly, Lu et al. focused on the research of 2D materials with main group elements and provided a detailed summary of their research progress in phase, synthesis, characterization, and applications.^[^ ^33^ ^]^ Additionally, Wang et al. summarized the design methods of 2D materials, nanoclusters, and single‐atom catalysts for improving the catalytic activity of CO_2_RR, including electrocatalysis and photocatalysis.^[^ ^34^ ^]^ Compared to these reported reviews focusing on similar areas, our review covers a broader and more systematic range of topics. This includes comprehensive types of 2D heterostructures (e.g., 2D/0D, 2D/1D, 2D/2D, and 2D/3D) as well as energy conversion applications (e.g., HER, OER, OWS, CO_2_RR, NRR). Thus, this comprehensive review will be favorable for designing heterostructures based on 2D materials with superior performance and development potential.

## 2 Water Splitting

Nowadays, issues associated with the greenhouse effect have increased people's awareness of environmental protection. Researchers are also dedicated to the development of new energy. Hydrogen, as a clean energy source, possesses a reaction enthalpy of −572 KJ mol^−1^. The energy density of H_2_ is remarkably high, making it a promising alternative to fossil fuels.^[^ ^128^, ^129^ ^]^ Various methods are employed to generate hydrogen energy, including electrolysis of water, hydrogen production from mineral fuels, and hydrogen production from biomass.^[^ ^130^ ^]^ Among these methods, electrolysis of water is an ideal way to produce hydrogen energy using surplus renewable energy.^[^ ^131^ ^]^ Furthermore, this method exhibits vast application prospects and a relatively mature preparation process. The reaction process involves HER and OER. Thus, the development of catalysts for OER and HER is a major impetus to boost progress in water electrolysis technology.^[^ ^132^ ^]^ During the water electrolysis reaction, catalysts are essential for reducing the reaction's overpotential and overcoming kinetic energy barriers.^[^ ^133^ ^]^ While noble metal catalysts demonstrate high efficiency, their scarcity and high cost pose challenges in meeting the demands of large‐scale hydrogen production.^[^ ^134^ ^]^

To meet the requirements of practical production, extensive studies have been conducted on low‐cost 2D materials with abundant resources. Heterostructures formed by these materials have become one of the most significant strategies to enhance catalytic performance in electrochemical reactions.^[^ ^135^ ^]^ The formation of heterostructures helps expose more catalytically active sites, improves electron mobility, and thus results in a higher exchange current density, lower Tafel slope, and reduced overpotential. Consequently, this enhances the electrocatalytic efficiency of the material.^[^ ^136^ ^]^ In this section, synthesis methods of heterostructures based on 2D materials and their advantages in electrolytic water splitting are introduced in detail, including HER, OER, and OWS.

### 2.1 HER

According to different reactions, HER can be achieved in two steps. The first step is the Volmer reaction. In an acidic environment, electrons combine with hydronium ions (H_3_O^+^) to generate adsorbed hydrogen atoms, while in alkaline solutions, hydroxide ions (OH^−^) and adsorbed hydrogen atoms are generated.^[^ ^137^ ^]^ In the second reaction step, if two adsorbed hydrogen atoms combine to form a hydrogen molecule for desorption, it is expressed as the Tafel step. On the other hand, if electrons first couple with protons and then combine with the adsorbed hydrogen atoms in the first step (the Volmer reaction) to generate desorbed hydrogen, it is expressed as the Heyrovsky step.^[^ ^138^ ^]^ In the Volmer reaction, the Tafel slope $b\;=\;\frac{2.3RT}{\alpha F}\;\approx \;120\;\mathrm{mV}$, where *R* represents the ideal gas constant, *T* represents the absolute temperature, *α* represents the symmetry coefficient of about 0.5, and *F* represents the Faraday constant.^[^ ^139^ ^]^ In the Tafel reaction, the slope is $b\;=\;\frac{2.3RT}{(1+\alpha )F}\;\approx \;40\;\mathrm{mV}$, while in the Heyrovsky reaction, it is $b\;=\;\frac{2.3RT}{2F}\;\approx \;30\;\mathrm{mV}$.^[^ ^140^ ^]^ The temperature of the aforementioned reactions is 25 °C. The Tafel slope is an inherent characteristic of the catalyst that expresses the current‐potential relationship and describes reaction kinetics.^[^ ^141^ ^]^ The formula is$\;\vartheta \;=\;b\;\log(\frac{j}{j_{0}})$, where *b* represents the Tafel slope, *j*_0_ is the exchange current density, *j* is the current density, and ϑ is the overpotential.^[^ ^142^ ^]^ The type of reaction (Heyrovsky reaction or Tafel reaction) can be inferred from the magnitude of the Tafel slope.

In an alkaline solution, the reaction rate is usually 2–3 orders of magnitude lower than in an acidic environment due to the decomposition of water, producing hydroxide ions, and adsorbed hydrogen atoms.^[^ ^143^ ^]^ The exchange current density of the hydrogen evolution reaction and the Gibbs adsorption free energy of hydrogen atoms (Δ*G*_H_) on the catalyst surface needs to satisfy the Sabatier rule, and the relationship diagram is similar to that of a volcano.^[^ ^144^ ^]^ Too high or too low adsorption‐free energy will decrease the exchange current density, leading to high overpotentials and reduced catalytic efficiency. Thus, when the value of Δ*G*_H_ approaches zero, the corresponding HER electrocatalysts are satisfactory.^[^ ^145^ ^]^ Commercial Pt has a Δ*G*_H_ of 0.09 eV, which is the most moderate, and its Tafel slope is 30 mV dec^−1^.^[^ ^146^ ^]^ Moreover, heterostructures formed by Pt and other materials can improve catalytic efficiency.^[^ ^147^ ^]^ For instance, Subbaraman et al. formed a heterostructure by growing Ni(OH)_2_ clusters on the surface of Pt that had been modified, which improved the catalytic efficiency of HER in an alkaline environment. Regarding its catalytic activity, the role of Pt's suitable Δ*G*_H_ was to improve the exchange current density, while Ni(OH)_2_ was responsible for providing a multitude of active sites for water splitting under alkaline conditions and enhancing its catalytic efficiency.^[^ ^148^ ^]^

#### 2.1.1 HER on 2D–0D Heterostructures

Among 2D–0D heterostructures, ensuring the uniform distribution of nanoparticles (NPs) on 2D materials without aggregation is crucial for exposing a massive number of active sites and maintaining good contact. Graphene oxide (GO), due to the abundant functional groups on its 2D sheets, facilitates chemical/electronic coupling with other materials, thereby achieving a uniform distribution of nanoparticles. MoS_2_ as a typical TMD material, exhibits a moderate adsorption‐free energy for hydrogen atoms (∆*G*_H_ of about 0.08 eV). However, the catalytic performance of bulk MoS_2_ in HER is not as good as that of MoS_2_ monolayers and nanodots.^[^ ^149^ ^]^ When MoS_2_ NPs are combined with reduced graphene oxide (rGO) to form a heterostructure electrocatalyst, the interaction, and synergistic effect can be fully utilized. For example, Li et al. prepared a MoS_2_/rGO heterostructure through a solvothermal reaction. The resulting MoS_2_ NPs were uniformly distributed on rGO sheets, exposing a large number of active sites. This led to excellent performance, including an overpotential of 0.1 V in an acidic solution, and a Tafel slope of 41 mV dec^−1^. The superior performance can be attributed to the fast electron transfer rate from MoS_2_ to the electrode, facilitated by the electrical coupling between MoS_2_ and graphene.^[^ ^35^ ^]^

**Figure 3:**
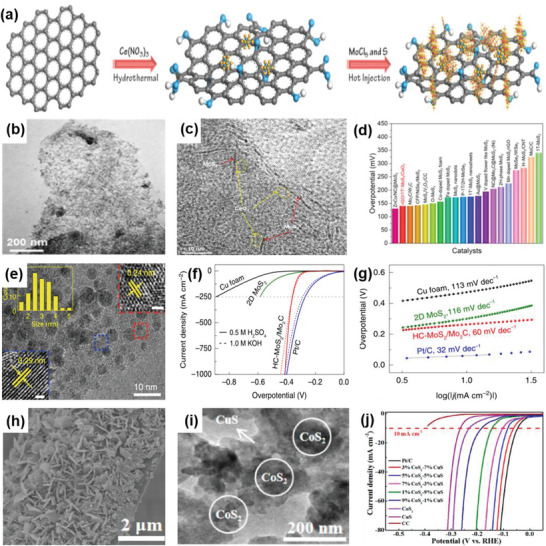
a) Schematic diagram of the preparation of rGO/1Tʹ‐MoS_2_/CeO_2_ heterostructure. b) TEM and c) HRTEM images of rGO/1Tʹ‐MoS_2_/CeO_2_. d) Overpotential of rGO/1T′‐MoS_2_/CeO_2_ at 10 mA cm^−2^ in 0.5 m H_2_SO_4_ compared with some Mo‐based HER electrocatalysts. Reproduced with permission.^[^ ^24^ ^]^ Copyright 2022, American Chemical Society. e) TEM image of MoS_2_/MoC_2_ heterostructure. f) LSV polarization curves and g) Tafel plots of MoS_2_/MoC_2_ and other samples in 0.5 m H_2_SO_4_. Reproduced with permission.^[^ ^36^ ^]^ Copyright 2020, Springer Nature. h,i) SEM and TEM images of the CoS_2_/CuS heterostructure. j) LSV polarization curves of CoS_2_/CuS and other catalysts in 0.5 m H_2_SO_4_. Reproduced with permission.^[^ ^38^ ^]^ Copyright 2019, American Chemical Society.

Furthermore, it has been demonstrated that 1Tʹ‐phase dominated TMD materials are promising candidates for HER catalysts.^[^ ^22^ ^]^ For example, Nie et al. successfully fabricated an rGO/1Tʹ‐MoS_2_ monolayers/CeO_2_ NPs heterostructure for HER through a wet‐chemical synthesis method (**Figure** **3**a–d). The CeO_2_ NPs tune the energy band structure of 1Tʹ‐MoS_2_, enhancing the HER activity. The synergistic effect of this 2D–0D heterostructure results in superior HER performance, including a Tafel slope of 43 mV dec^−1^ and good stability over 10 000 cycles.^[^ ^24^ ^]^ Moreover, 2D MoS_2_ nanosheets are commonly used as supports for preparing the heterostructure catalysts for HER. To improve the electrocatalytic performance of the regular MoS_2_ nanosheets, Chen et al. successfully converted porous MoS_2_ into nanoislands through a one‐step hydrothermal process. This morphology of MoS_2_ provides more effective edge active sites and enhances the inherent conductivity, leading to remarkable HER/OER bifunctional electrocatalytic performance.^[^ ^150^ ^]^ Similarly, Zhang et al. successfully prepared 2D MoS_2_ catalysts modified with 0D Mo_2_C NPs through an exfoliation method and a thermal treatment (Figure 3e–g). The thermal treatment enhances the mechanical robustness of the catalysts and offers effective electrical contact, resulting in improved catalytic performance, including an overpotential of 412 mV at a very high current density of 1000 mA cm^−2^ and a low Tafel slope of 60 mV dec^−1^. Additionally, this work has achieved mass production, which is of vital significance for practical applications.^[^ ^36^ ^]^

As the reduction in catalyst size improves catalytic performance, single‐atom catalysts have become a research hotspot in recent years. This involves the presence of all metal components on the carrier in the form of a monoatomic dispersion, without any homoatomic metal–metal bonds. Therefore, it is essential to highlight that the heterostructure formed by MoS_2_ nanosheets and single atoms significantly enhances the HER performance. For example, Deng et al. prepared 0D–2D Pt‐MoS_2_ heterostructure electrocatalysts, in which single Pt atoms are uniformly dispersed on the MoS_2_ plane using a one‐pot synthesis method. The density functional theory (DFT) results have proven that the adsorption of Pt atoms could not only adjust the adsorption behavior of H atoms on adjacent S sites but also enhance the activity for HER. As a result, compared with pure MoS_2_, the electrocatalytic activity and stability of the Pt‐MoS_2_ heterostructure in an acidic medium have been greatly improved.^[^ ^37^ ^]^

Considering the issue of the high cost, the combination of non‐noble metal‐based catalysts in the form of 2D–0D heterostructures has introduced an alternative route to attain excellent catalytic performance. For instance, Li et al. prepared a catalyst composed of CuS nanosheets decorated with CoS_2_ NPs using the hydrothermal‐electrodeposition method (Figure 3h–j). Due to the regulatory effect of the heterostructure on electrons and energy bonds, the CuS/CoS_2_ heterostructure exhibited a low Tafel slope of 46 mV dec^−1^ and a small overpotential of 62 mV at 10 mA cm^−2^ in 0.5 m H_2_SO_4_. It also demonstrated good catalytic activity in 0.5 m phosphate buffer aqueous solutions and 1 m KOH.^[^ ^38^ ^]^ However, dangling bonds, poor‐quality interfaces, and crystal lattice dislocations continue to pose challenges in the synthesis process of the 2D–0D heterostructure.^[^ ^151^ ^]^ Thus, designing a rational morphological structure and optimizing catalytic performance in the future still require further exploration.

#### 2.1.2 HER on 2D–1D Heterostructures

**Figure 4:**
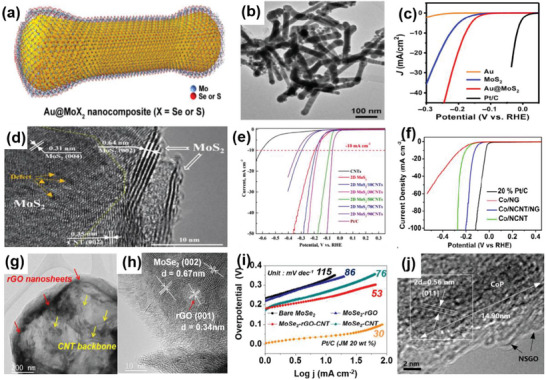
a) Schematic illustration of Au@MoS_2_ heterostructure. b) TEM image of Au@MoS_2_. c) Polarization curves of Au@MoS_2_ and corresponding samples in 0.5 m H_2_SO_4_ solution. Reproduced with permission.^[^ ^39^ ^]^ Copyright 2019, Springer Nature. d) HRTEM image of 2D–1D MoS_2_/CNTs heterostructure; e) LSV curves of MoS_2_/CNTs heterostructure and other compared catalysts in 0.5 m H_2_SO_4_. Reproduced with permission.^[^ ^40^ ^]^ Copyright 2023, Small. f) The HER polarization curves of Co/NCNT/NG heterostructure and 20% Pt/C in 0.5 m H_2_SO_4_ solution. Reproduced with permission.^[^ ^41^ ^]^ Copyright 2018, Royal Society of Chemistry. g) TEM and h) HRTEM images of MoSe_2_–rGO–CNT heterostructure. i) Tafel plots of MoSe_2_–rGO–CNT heterostructure. Reproduced with permission.^[^ ^42^ ^]^ Copyright 2017, American Chemical Society. j) HRTEM image of CoP/NSGO. Reproduced with permission.^[^ ^43^ ^]^ Copyright 2016, Elsevier Ltd.

The morphology and structure of 1D materials mainly include nanowires, nanotubes, and nanofibers. Generally, 1D materials have a high aspect ratio, which is more conducive to electron transmission, while 2D materials provide good support for the 1D materials. Compared with 2D–0D materials, the specific surface area of 2D–1D materials is larger, resulting in a higher density of active sites. These advantages enable the 2D–1D structure to have a better synergistic effect on the heterostructure. For instance, Liu et al. successfully prepared an Au nanorods@MoS_2_ monolayer heterostructure using a colloidal synthesis approach (**Figure** **4**a–c). Benefiting from the effective electron transfer between these two materials, this 2D–1D heterostructure showed outstanding HER performance with a potential of 178 mV at 10 mA cm^−2^ and a Tafel slope of 43.3 mV dec^−1^.^[^ ^39^ ^]^

Carbon nanotubes (CNTs) are tubular structures formed by the sp^2^ hybridization of carbon atoms (with a small amount of sp^3^ hybridization). They possess a high aspect ratio, generally above 1000:1, and can be categorized into single‐walled carbon nanotubes (SWCNTs) and multiwalled carbon nanotubes (MWCNTs) based on their number of layers. In comparison to MWCNTs, SWCNTs have fewer defects, resulting in excellent electronic and mechanical properties. However, mass production of SWNTs remains challenging, leading to most carbon nanotube preparation methods centering around MWCNTs. Researchers have summarized several preparation methods for CNTs and introduced their applications in composite materials, field emission, electronics, and electrochemistry.^[^ ^152^, ^153^ ^]^ CNTs can be utilized in electrocatalytic HER in combination with other materials, forming heterostructures that enhance catalytic activity and conductivity, with remarkable progress being made.^[^ ^154^, ^155^, ^156^, ^157^, ^158^, ^159^, ^160^ ^]^ For example, Yang et al. successfully combined 2D MoS_2_ with 1D CNTs using the liquid‐phase synthesis approach. The MoS_2_ nanosheets were attached to the surface of CNTs (Figure 4d,e). The introduction of CNTs enhanced the conductivity of S‐vacancy‐contained MoS_2_, thereby improving electron mobility and catalytic activity. Due to the synergistic effect, the hydrogen adsorption capacity of this 2D–1D heterostructure was improved, resulting in favorable HER performance comparable to that of commercial Pt/C.^[^ ^40^ ^]^ Similarly, Cao et al. mixed g‐C_3_N_4_ and Co salt in different proportions and pyrolyzed them to synthesize a Co, N‐codoped CNT‐graphene heterostructure. Due to the presence of rich catalytic active sites, Co/NCNT/NG exhibited remarkable catalytic activity for HER with an overpotential of 123 mV at 10 mA cm^−2^ under acidic conditions (Figure 4f).^[^ ^41^ ^]^ Another example is the work of Park et al. who prepared a 2D–1D MoSe_2_–rGO–CNT heterostructure using a spray pyrolysis process. The high aspect ratio of CNTs helped in forming a porous spherical framework, reducing the accumulation of rGO nanosheets (Figure 4g–i). CNTs and rGO nanosheets also inhibited the growth of MoSe_2_ nanocrystals, leading to a structure containing ultrafine MoSe_2_ nanocrystals. This ternary heterostructure exhibited a higher catalytic efficiency than binary materials, with a relatively low overpotential of 0.24 V at 10 mA cm^−2^ and a Tafel slope of 53 mV dec^−1^. The synergy effect and porous structure also made it suitable for use in sodium‐ion batteries.^[^ ^42^ ^]^

The most common 2D–1D heterostructure models involve 1D materials arranged parallel to 2D substrates and perpendicular to 2D materials. This parallel arrangement provides a high contact area, strengthening the interaction during the catalytic reaction. For instance, Lin et al. reported a thermal decomposition method to prepare 1D CoP nanorods in situ grown on 2D N, S co‐doped graphene oxide (Figure 4j). The synergistic effect from heterostructure and the larger electroactive area provided by N, S co‐doping can promote the CoP/NSGO material to obtain excellent HER performance, including an overpotential of 45 mV at 10 mA cm^−2^ and a Tafel slope of 52 mV dec^−1^.^[^ ^43^ ^]^

Although heterostructure catalysts described above are simple to prepare, these structures require the transfer of electrons between layers, resulting in high electrical resistance values. The preparation of heterostructures with 1D material perpendicular to the 2D substrate allows for the full utilization of the high aspect ratio of the 1D material, enabling faster electron transport in a tubular channel. For instance, Tang et al. synthesized a 2D–1D heterostructure of Ni_2_P nanosheets@CoP nanowires as electrocatalysts for HER. Such a heterostructure possessed electronic interactions and a synergistic effect, which were beneficial for enhancing the catalytic activity of HER. As expected, Ni_2_P@CoP exhibited remarkable performance compared to traditional metal phosphides (TMPs) catalysts, with an overpotential of only 55 mV at 10 mA cm^−2^ and a Tafel slope of 48 mV dec^−1^.^[^ ^44^ ^]^

#### 2.1.3 HER on 2D–2D Heterostructures

**Figure 5:**
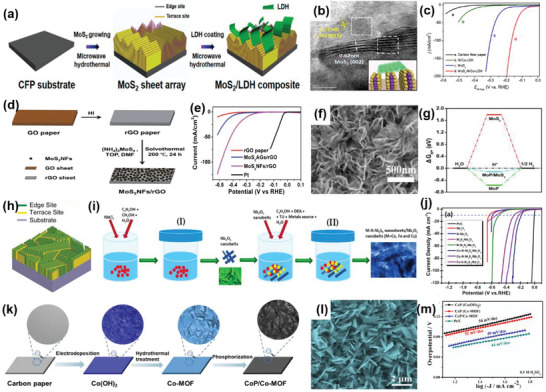
a) Schematic diagram of the fabrication process of MoS_2_/NiCo–LDH. b) HRTEM image of the MoS_2_/NiCo–LDH. c) HER polarization curves of MoS_2_/NiCo–LDH and other catalysts in 1 m KOH solution. Reproduced with permission.^[^ ^45^ ^]^ Copyright 2017, Elsevier Inc. d) Schematic for the formation of MoS_2_ NF/rGO heterostructure. e) Polarization curves of MoS_2_ NF/rGO heterostructure. Reproduced with permission.^[^ ^46^ ^]^ Copyright 2014, Royal Society of Chemistry. f) SEM images of MoP/MoS_2_. g) HER free‐energy diagram MoP/MoS_2_. Reproduced with permission.^[^ ^47^ ^]^ Copyright 2019, American Chemical Society. h) Idealized structure of vertically grown molybdenum chalcogenide films. Reproduced with permission.^[^ ^48^ ^]^ Copyright 2013, American Chemical Society. i) Schematic formation of M–N–Ni_9_S_8_/Nb_2_O_5_ heterostructure (M = Co, Fe, or Cu). j) Polarization curves M–N–Ni_9_S_8_/Nb_2_O_5_ heterostructure. Reproduced with permission.^[^ ^49^ ^]^ Copyright 2021, Elsevier B.V. k) Schematic illustration for the synthesis of CoP/Co–MOF heterostructure. l) SEM image of CoP/Co–MOF. m) Tafel slopes of CoP/Co–MOF and other samples in 0.5 m H_2_SO_4_. Reproduced with permission.^[^ ^50^ ^]^ Copyright 2021, Elsevier B.V.

2D–2D heterostructures can be categorized into two types: those perpendicular to the substrate plane and those parallel to it. Since the catalytically active sites of MoS_2_ are located at the edges of the layers, a vertical arrangement exposes numerous active sites. Consequently, the heterostructure formed by the vertically arranged 2D MoS_2_ exhibits a significant increase in catalytic activity in HER. For instance, Hu et al. prepared a 2D–2D MoS_2_/Ni‐Co layered double hydroxide (LDH) heterostructure (**Figure** **5**a) using the hydrothermal method. Figure 5b clearly illustrates that the heterostructure plane is aligned perpendicular to the base material. The adsorption of LDHs on the hydroxyl group enables faster decomposition of water molecules, resulting in excellent performance of the obtained heterostructure, with an overpotential of 78 mV at 10 mA cm^−2^ and a Tafel slope of 76.6 mV dec^−1^ in alkaline conditions (Figure 5c).^[^ ^45^ ^]^ Ma et al. successfully coated MoS_2_ nanoflowers (NFs), composed of nanosheets, on rGO through the solvothermal method (Figure 5d,e). MoS_2_ NFs uniformly cover the surface of rGO. Due to the abundance of active catalytic edges on uniform MoS_2_ NSs, MoS_2_ NF/rGO exhibited outstanding performance toward HER, with a low overpotential at −0.19 V at 10 mA cm^−2^ and a Tafel slope of 95 mV dec^−1^.^[^ ^46^ ^]^ Wu et al. designed the 2D–2D MoP/MoS_2_ heterostructure as the catalyst for HER (Figure 5f,g). The 2D nanosheets provide rich active sites for H_2_O activation, while MoP, with its high conductivity, and the interface between MoP and MoS_2_ promoted electron transfer rate. Thus, MoP/MoS_2_ heterostructure exhibits excellent performance in HER, including Δ*G*_H_ value approaching zero according to DFT calculations and an overpotential of around 96 mV at 10 mA cm^−2^. Moreover, under both the neural and alkaline conditions, the MoP/MoS_2_ heterostructure showed more remarkable HER properties at high current density compared to the Pt/C catalyst.^[^ ^47^ ^]^ Kong et al. adopted a CVD method to synthesize MoS_2_ or MoSe_2_ films vertically on different substrates (Figure 5h). The author believed that the utilization of sulfur powder instead of hydrogen sulfide (H_2_S) was the main reason for vertical growth. The vertically grown heterostructure exposed a significant amount of layered edges. Furthermore, since the charge is transported within the layers instead of between the layers, the obtained heterostructure exhibited high electrical conductivity, which was beneficial for the HER.^[^ ^48^ ^]^

For materials with catalytically active sites solely on the outermost layer, 2D–2D heterostructures parallel to each other can significantly enhance the electrolyte contact area. Meanwhile, the energy band of the catalyst can be adjusted by the substrate. For example, Chandrasekaran et al. fabricated a heterostructure of 2D–2D M–N–Ni_9_S_8_ nanosheets/Nb_2_O_5_ nanobelts (M = Co, Fe, or Cu) using a two‐step hydrothermal method (Figure 5i,j). Among the tested heterostructures for HER electrocatalyst, including Nb_2_O_5_ NBs, Ni_9_S_8_/Nb_2_O_5_, Fe–N–Ni_9_S_8_/Nb_2_O_5_, Co–N–Ni_9_S_8_/Nb_2_O_5_, and Cu–N–Ni_9_S_8_/Nb_2_O_5_, the Co–N–Ni_9_S_8_/Nb_2_O_5_ catalyst demonstrated the lowest overpotential of −171 mV versus RHE at 10 mA cm^−2^ and the smallest Tafel slope of 69 mV dec^−1^ under acidic conditions. The DFT results suggested the importance of metals Co‐doping with N atoms, which significantly affected the modulation of local charge distribution and electronic properties at the interface between Ni_9_S_8_ nanosheets and Nb_2_O_5_ nanobelts.^[^ ^49^ ^]^

In another example, Li et al. employed three approaches, including electrodeposition, hydrothermal treatment, and partial phosphorization, to synthesize CoP/Co–MOF nanosheets. These nanosheets were then applied as an electrocatalyst for HER (Figure 5k–m). This work illustrated that synthesis time and temperature can affect the amount of CoP/Co–MOF interfacial sites, thus further influencing the HER activity. When the phosphorization temperature and time were set at 320 °C and 2 h, the resulting CoP/Co–MOF with suitable interfacial sites between CoP and Co–MOF catalyst exhibited superiority over other corresponding catalysts synthesized under different conditions. This superiority was attributed to the presence of suitable interfacial sites between CoP and Co–MOF. This CoP/Co–MOF heterostructure, which exhibited the best HER activity, showed overpotentials of 52, 106, and 206 mV at 10 mA cm^−2^ in acidic, neutral, and alkaline conditions, respectively. Across the wide range of pH levels, the CoP/Co–MOF catalyst demonstrated not only excellent activity but also satisfactory durability.^[^ ^50^ ^]^

**Figure 6:**
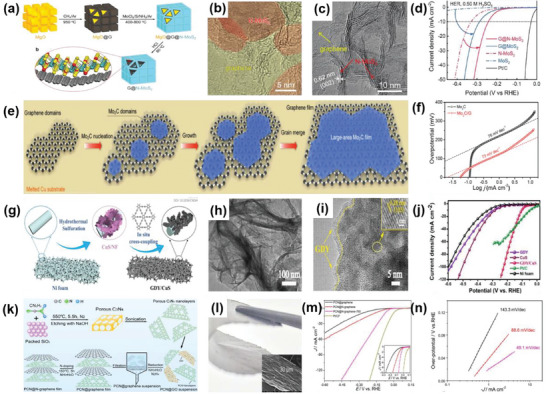
a) Schematic illustration of the preparation of G@N–MoS_2_ heterostructure. b,c) HRTEM images of G@N–MoS_2_. d) HER polarization curves of G@N–MoS_2_ and other samples. Reproduced with permission.^[^ ^51^ ^]^ Copyright 2017, Wiley‐VCH. e) Schematic showing the growth of Mo_2_C crystals grown on graphene. f) Tafel plots of Mo_2_C and Mo_2_C/graphene. Reproduced with permission.^[^ ^52^ ^]^ Copyright 2017, Wiley‐VCH. g) Schematic illustration for the preparation of CuS/GDY heterostructure. h) TEM and i) HRTEM images of GDY/CuS. j) Polarization curves of GDY/CuS and other catalysts in 1.0 m KOH. Reproduced with permission.^[^ ^53^ ^]^ Copyright 2019, Royal Society of Chemistry. k) The preparation process of PCN@N–graphene heterostructure. l) Photo of PCN@N–graphene film (inset: SEM image of the cross‐section view of PCN@N–graphene). m) LSV curves, n)Tafel plots of PCN@N–graphene. Reproduced with permission.^[^ ^54^ ^]^ Copyright 2015, American Chemical Society.

Graphene is a material entirely made up of carbon, composed of single‐atom‐thick layers with sp and sp^2^ hybrid carbon networks. Graphene possesses satisfactory electronic conductivity, and its planar porous structure facilitates electrolyte diffusion, mass transfer, and gas release. This makes graphene a promising substrate for growing other 2D materials. For example, Tang et al. fabricated a porous 2D–2D N‐doped MoS_2_/graphene heterostructure using a template method (**Figure** **6**a), which can serve as a trifunctional catalyst. Figure 6b,c clearly indicates the formation of a layer‐by‐layer stacking structure. MoS_2_ can improve HER activity, while graphene can enhance OER and ORR activity. As a result, it exhibited good HER performance, including an overpotential of 243 mV at 10 mA cm^−2^ and a Tafel slope of 82.5 mV dec^−1^, outperforming the MoS_2_ material alone (Figure 6d). Importantly, the heterostructure also exhibited outstanding catalytic properties for OER and ORR in an alkaline environment.^[^ ^51^ ^]^ Moreover, Geng et al. utilized a CVD process to grow large‐area Mo_2_C films on graphene (Figure 6e). Graphene can limit the diffusion rate of Mo, making it easier for Mo_2_C to form a hexagonal structure on the edge of the graphene with high crystallinity. The electronic coupling between graphene and Mo_2_C was beneficial for enhancing catalytic efficiency. As expected, the prepared heterostructure showed excellent performance, including an overpotential of 236 mV at 10 mA cm^−2^ and a Tafel slope of 73 mV dec^−1^ under acidic conditions (Figure 6f).^[^ ^52^ ^]^ In addition, Shi et al. prepared a GDY/CuS heterostructure in which graphdiyne coated CuS nanosheets on nickel foam (NF) served as an HER catalyst (Figure 6g). This GDY/CuS heterostructure exhibited a thin flake morphology with a wrapping structure (Figure 6h,i). Thanks to the close interaction, this heterostructure demonstrated outstanding HER performance achieving an overpotential of 106 mV at 10 mA cm^−2^ (Figure 6j). Moreover, this work indicates that CuS nanosheets can be used not only as an HER catalyst but also as a substrate for the in situ growth of graphdiyne.^[^ ^53^ ^]^

Regulating the elemental composition and structure is a common strategy to improve the performance of HER electrocatalysts. The defects and edges of g‐C_3_N_4_ are important catalytic centers for HER, while the generation of in‐plane pores introduces additional edges and defects in 2D C_3_N_4_ nanolayers. For example, Duan et al. prepared a porous C_3_N_4_ (PCN)@N‐graphene film through a vacuum filtration method (Figure 6k,l). This PCN@N‐graphene catalyst possessed a strong synergistic effect and highly exposed active sites, which benefited the HER performance. Consequently, the electrocatalyst exhibited extraordinary HER activity, including a low overpotential of 80 mV and a Tafel slope of 49.1 mV dec^−1^ (Figure 6m,n).^[^ ^54^ ^]^

#### 2.1.4 HER on 2D–3D Heterostructures

When forming 2D–3D heterostructures, it is usually difficult to achieve surface‐to‐surface contact in the 2D–3D heterostructure due to the significant difference in surface energy at different positions of the 3D structure. Therefore, 2D materials mostly adopt a hierarchical structure to grow vertically on the surface of a 3D substrate, allowing electrons to be rapidly transported in the 2D plane. The hierarchical structure ensures an increased contact area with the electrolyte, promoting good contact between the substrates. Simultaneously, the 3D substrate structure (such as carbon fiber, porous foam, nanocage, etc.) allows the electrolyte to penetrate its interior and enlarge the specific surface area. For instance, Miao et al. prepared 2D Ni–Mo–S nanosheets grown on a 3D carbon fiber surface for use as a high‐efficiency electrocatalyst for HER. The introduction of Ni not only created rich defect sites but also adjusted the morphology and inherent properties of MoS_2_ on carbon fibers. As a result, compared to MoS_2_/C, the Ni–Mo–S/C electrocatalyst exhibited better HER performance with an overpotential of 200 mV at 10 mA cm^−2^ and a Tafel slope of 85.3 mV dec^−1^.^[^ ^55^ ^]^ In another case, Zhang et al. used a dissolution‐regrowth and subsequent phosphorization pathway to prepare a heterostructure with 3D CoP·5CoMoP nanocages, composed of 2D CoP and CoMo nanosheets. CoP·5CoMoP nanocages possessed a hollow structure wrapped by nanosheets. Furthermore, the results showed that the hydrogen adsorption values of the P‐ and Co‐sites were 0.32 and −0.3 eV, respectively, which were close to zero. Thus, CoP·5CoMoP catalysts for HER exhibited outstanding performance and worked within a broad pH range.^[^ ^56^ ^]^

**Figure 7:**
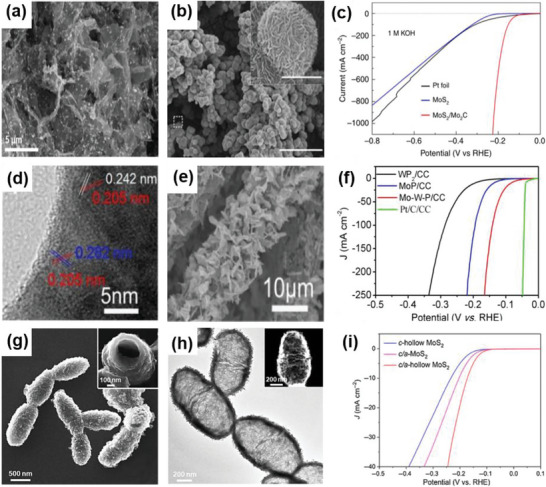
a) SEM image of H–2D/3D–MoS2–rGO heterostructure. Reproduced with permission.^[^ ^57^ ^]^ Copyright 2021, MDPI. b) SEM image of the MoS_2_/Mo_2_C heterostructure. c) Polarization curves of the MoS_2_/Mo_2_C heterostructure and other catalysts. Reproduced with permission.^[^ ^59^ ^]^ Copyright 2019, Springer Nature. d,e) HRTEM and SEM images of Mo–W–P/CC. f) Polarization curves of Mo–W–P/CC and other catalysts. Reproduced with permission.^[^ ^60^ ^]^ Copyright 2016, Royal Society of Chemistry. g,h) SEM and TEM images of MoS_2_ hollow structure. i) Polarization curves of MoS_2_ hollow structures. Reproduced with permission.^[^ ^11^ ^]^ Copyright 2022, Chinese Chemical Society.

Although 2D MoS_2_ is a good candidate for HER activities, it usually lacks a sufficient number of undercoordinated edge atoms. To solve this problem, Choi et al. synthesized hierarchical MoS_2_ nanosheets on a 3D rGO aerogel to form a 2D–3D MoS_2_–rGO heterostructure through a one‐pot hydrothermal method (**Figure** **7**a). The 3D porous structure of rGO produced a relatively large surface area and abundant edge sites, leading to a remarkable electrochemical performance in HER.^[^ ^57^ ^]^ In another example, Tan et al. demonstrated that introducing lattice strains is an effective route to promote the catalytic performance of MoS_2_. With its porous and curved structure, the electrocatalytic activity of 2D MoS_2_ was activated by introducing out‐of‐plane strains. Due to its specific particular electronic properties and the low‐resistance Ohmic contact between the curved monolayer MoS_2_ and the NPG substrate, the electrode performed high electrocatalytic activity, characterized by an onset potential of −118 mV and a Tafel slope of 46 mV dec^−1^.^[^ ^58^ ^]^ Furthermore, Luo et al. fabricated MoS_2_ microspheres composed of MoS_2_ NSs decorated with Mo_2_C NPs through a hydrothermal and CVD method (Figure 7b,c). This spherical morphology is beneficial for both the access of reactants and the release of H_2_. Moreover, Mo_2_C can improve the performance of HER by altering the surface chemistry of MoS_2_. Thus, the MoS_2_/Mo_2_C electrocatalysts demonstrated remarkable pH‐universal performance in HER, exhibiting a low overpotential of 227 mV at a high current density of 1000 mA cm^−2^ and a low Tafel slope of 53 mV dec^−1^ under acidic conditions. Additionally, under alkaline conditions, they showcased a low overpotential of 220 mV at 1000 mA cm^−2^ and a low Tafel slope of 44 mV dec^−1^.^[^ ^59^ ^]^ Wang et al. synthesized nanosheets of molybdenum tungsten phosphide (Mo‐W‐P) on carbon cloth (CC) catalysts, achieving an interplanar distance of 0.242 nm, through a hydrothermal and phosphatization method (Figure 7d–f). Nanosheets are uniformly distributed on the fibers of CC. Due to the strong synergistic effect of W and Mo atoms, the porous heterostructure of Mo‐W‐P/CC showed superior performance in HER. Specifically, the electrocatalyst required a low overpotential of 138 mV to drive an ultrahigh current density of 100 mA cm^−2^ under acid conditions.^[^ ^60^ ^]^ In addition, Liu et al. reported the synthesis of a 3D hollow structure formed through the solvothermal method, where a 2D crystalline/amorphous MoS_2_ nanosheet self‐assembled (Figure 7g–i). This unique hollow structure, consisting of both crystalline and amorphous regions, was conducive to the exposure of more active sites. As a result, this heterostructure showed excellent HER performance, including a Tafel slope of 45 mV dec^−1^ and outstanding stability of 10 000 cycles.^[^ ^11^ ^]^

Utilizing MOFs as precursors to fabricate functional heterostructures based on transition metals and carbon can be a valuable approach for producing catalysts with enhanced mass transfer ability and rich active sites. For example, Qin et al. utilized a facile hydrothermal method to grow MoS_2_ nanosheets on Co_3_O_4_ nanoleaves. The strong electrostatic interaction between the positively charged Co_3_O_4_ nanoleaves and the negatively charged MoS_2_ nanosheets facilitated the construction of these heterostructures. The different valence states of Co and the multitude of active sites provided by the heterostructure effectively enhanced the catalytic activity for HER. At a current density of 20 mA cm^−2^, the catalyst showed an overpotential of 205 mV for HER and a small Tafel slope of 98 mV dec^−1^ under the 1 m KOH condition.^[^ ^61^ ^]^

### 2.2 OER

The dominant limitation in the water‐splitting process arises from the high overpotential for OER, which is attributed to the involvement of four electrons and the formation of covalent O─O bonds.^[^ ^161^, ^162^ ^]^ The Tafel slope of the OER is calculated as $b\;=\;\frac{2.3RT}{\alpha F}$, where *b* represents the Tafel slope, *R* denotes the ideal gas constant, *T* represents the absolute temperature, α is the transfer coefficient, and *F* is the Faraday constant. There are also multiple possible paths for OER, each involving the transfer of four electrons during the OER process. The most common steps^[^ ^163^ ^]^ are as follows

(1) $$*+{\mathrm{OH}}^{-}\to *\mathrm{OH}+e^{-}$$

(2) $$*\mathrm{OH}\to *O^{•}+H^{+}+e^{-}$$

(3) $$*O^{•}+{\mathrm{OH}}^{-}\to *\mathrm{OOH}+e^{-}$$

(4) $$*\mathrm{OOH}\to *+O_{2}+H^{+}+e^{-}$$

Here * indicates the state of being adsorbed on the surface of the catalyst. The change in Gibbs free energy during each intermediate reaction is denoted by ∆*G*. Additionally, it can be observed from the volcano diagram that *∆G* hinders the progress of the reaction when it is too high or too low. Therefore, the closer ∆*G* is to zero, the stronger the catalytic effect. The catalytic performance is determined by the step with the highest ∆*G* among the four reaction steps.

Materials such as RuO_2_ and IrO_2_, with rutile structures, exhibit excellent OER activity in acidic and alkaline conditions.^[^ ^164^, ^165^ ^]^ Nevertheless, these two materials can be easily oxidized to RuO_4_ or IrO_3_ at high anode potentials, increasing the cost of electrolyzing water.^[^ ^161^, ^162^ ^]^ Hence, the development of non‐noble metal catalysts with properties superior to those of RuO_2_ and IrO_2_ is beneficial for OER applications.^[^ ^166^ ^]^ In recent years, an increasing number of heterostructures consisting of non‐noble metal materials have not only achieved excellent performance but also significantly reduced costs.

#### 2.2.1 OER on 2D–0D Heterostructures

**Figure 8:**
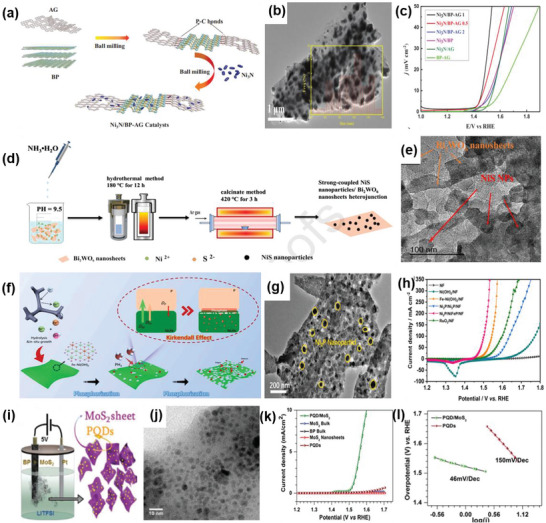
a) Schematic illustration of the fabrication of the Ni_3_N/BP–AG heterostructure. b) TEM image of the Ni_3_N/BP–AG heterostructure. c) OER polarization curves of the Ni_3_N/BP–AG heterostructures. Reproduced with permission.^[^ ^62^ ^]^ Copyright 2019, Wiley‐VCH. d) Schematic process for the preparation of NiS/Bi_2_WO_6_ heterostructure. e) TEM image of NiS/Bi_2_WO_6_. Reproduced with permission.^[^ ^63^ ^]^ Copyright 2020, Elsevier B.V. f) Schematic illustration of the formation of Ni_2_P/NiFeP/Ni heterostructure. g) TEM images of Ni_2_P/NiFeP/NF. h) OER LSVs of Ni_2_P/NiFeP/NF and other samples. Reproduced with permission.^[^ ^64^ ^]^ Copyright 2022, Elsevier B.V. i) Schematic showing the electrochemical synthesis of PQD/MoS_2_ heterostructure. j) High‐resolution TEM image of PQD/MoS_2_. k) LSVs acquired for determining the OER activities in 0.1 m KOH solutions. l) Tafel plots of PQD/MoS_2_ and PQDs. Reproduced with permission.^[^ ^65^ ^]^ Copyright 2020, Royal Society of Chemistry.

Similar to HER, forming a 2D–0D heterostructure as the OER electrocatalyst can increase the contact area between materials, enrich the number of active sites, and refine the catalytic efficiency of OER. The abundant functional groups on the surface of graphene oxide can interact with NPs, allowing for their uniform dispersion. For example, Wang et al. utilized an activated 2D graphene (AG)–black phosphorus (BP) nanosheet heterostructure to support 0D Ni_3_N NPs (Ni_3_N/BP–AG) through a simple mechanical ball milling method under argon protection (**Figure** **8**a–c). BP nanosheets exposed abundant edges and internal defects, which were conducive to increasing the quantity of catalytically active sites. As expected, the Ni_3_N/BP–AG heterostructure exhibited good catalytic activity, with an overpotential of 233 mV at 10 mA cm^−2^ and a Tafel slope of 42 mV dec^−1^. This mainly resulted from three factors: 1) BP possessing a suitable bandgap and good conductivity, 2) transition metal nitrides providing abundant active sites, and 3) AG playing a crucial role in stabilizing and connecting several materials.^[^ ^62^ ^]^ For another example, Li et al. synthesized a 0D–2D NiS/Bi_2_WO_6_ heterostructure electrocatalyst via a hydrothermal and calcined synthesis strategy (Figure 8d,e). The bonding of Ni and O changed the valence‐bond environment around the nickel species and also equilibrated the adsorption of OH^−^ and the desorption of O_2_. Thus, the catalyst exhibited good performance, with an overpotential of 527 mV at 10 mA cm^−2^ and a Tafel slope of 238 mV dec^−1^ for OER.^[^ ^63^ ^]^

It has also been noticed that TMPs can be promising catalysts for the OER due to their high activity, good stability, and low cost. For instance, Wu et al. prepared 0D Ni_2_P NPs and 2D NiFeP nanosheets on NF via in situ growth and phosphorization methods, which served as an OER electrocatalyst (Figure 8f–h). As possessing rich interfaces, the Ni_2_P/NiFeP/NF heterostructure offered efficient active sites for oxygen generation. Additionally, other structural features, like high pore volumes and specific surface area, were also advantageous factors facilitating catalytic activity.^[^ ^64^ ^]^ In addition to NPs, quantum dots (QDs) also provide catalytically active sites. For example, as displayed in Figure 8i,j, Prasannachandran et al. prepared 0D–2D heterostructures of PQD/MoS_2_. These heterostructures consisted of few‐layered MoS_2_ nanosheets and PQDs, with the PQDs interspersed well into the MoS_2_ nanosheets. Regarding the OER activity, this heterostructure catalyst showed an overpotential of 370 mV at 10 mA cm^−2^ and a Tafel slope of 46 mV dec^−1^ (Figure 8k,l).^[^ ^65^ ^]^

#### 2.2.2 OER on 2D–1D Heterostructures

**Figure 9:**
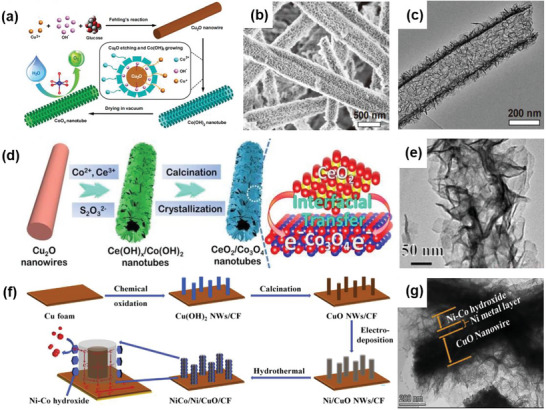
a) Fabrication procedure of the hierarchical CoO*_x_* heterostructure. b,c) SEM and TEM images of the constructed heterostructure. Reproduced with permission.^[^ ^66^ ^]^ Copyright 2015, Wiley‐VCH. d) Illustration of the synthesis process of the CeO_2_/Co_3_O_4_ heterostructure. e) TEM image of CeO_2_/Co_3_O_4_. Reproduced with permission.^[^ ^67^ ^]^ Copyright 2019, American Chemical Society. f) The synthesis process of NiCo/Ni/CuO core–shell nanoarrays on CF. g) TEM image of NiCo/Ni/CuO/CF heterostructure. Reproduced with permission.^[^ ^68^ ^]^ Copyright 2020, Elsevier Ltd.

The 1D nanostructures exhibit large specific surface areas and oriented electronic/ionic transport pathways. These advantages enable 2D–1D heterostructures to exhibit higher catalytic efficiency for OER. For example, Alia et al. reported that Ir–Ni and Ir–Co nanowires displayed significant activity advantages over their corresponding NPs catalysts. The OER mass activity of Ir–Ni nanowires was found to be 10 times higher than that of Ir NPs, while Ir–Co nanowires exhibited 9 times higher OER mass activity compared to Ir NPs.^[^ ^167^ ^]^ As a result, many studies on 2D‐based heterostructure for OER are now focusing more on 2D–1D heterostructures to achieve higher catalytic performance. For example, Wang et al. mimicked the structure of leaves and synthesized a 2D–1D CoO*_x_* nanosheet/nanotube heterostructure catalyst (**Figure** **9**a). Figure 9b,c indicates the formation of CoO*_x_* nanotubes with an external diameter of 200 nm. Thanks to the highly active Co^2+^ electronic structure, enlarged surface area, and enhanced conductivity, the catalyst showed excellent OER performance. It achieved a current density of 51.2 mA cm^−2^ at 1.65 V versus RHE and demonstrated a Tafel slope of 75 mV dec^−1^ for OER.^[^ ^66^ ^]^

Heterostructures formed by transition metal oxide (TMO) materials can serve as efficient electrocatalysts for OER. Among the TMOs commonly used as OER catalysts are Co_3_O_4_, CeO_2_, Fe_2_O_3_, MnO_3_, and others. For instance, the heterostructure formed by Co_3_O_4_ can significantly enhance the catalytic efficiency of OER. As an example, Lu et al. prepared a blend of oxide nanowire arrays of Co and Ni that were arranged in a casual manner on NF. Since chemical binders not only reduce the contact area between electrolytes and catalytic active sites but also lower the overall conductivity of the electrode, direct growth of catalysts on a metal foam substrate should be applied to eliminate the need for isolating chemical binders. The ratio of Ni to Co can influence the morphology, structure, and performance of the catalyst. An increase in Ni content enlarges its specific surface area, roughness factor, and conductivity.^[^ ^168^ ^]^

CeO_2_ also has broad application prospects in the field of electrocatalysis. The oxygen vacancy defects of CeO_2_ are conducive to obtaining a high oxygen storage capacity. When the adsorbed substance is oxidized on the surface, the oxidant usually is the surface lattice oxygen. A large number of lattice oxygen and oxygen vacancies in CeO_2_ are conducive to the adsorption and oxidation of the intermediate products during the OER process. For example, Li et al. obtained the CeO_2_–CoO nanofiber heterostructure with excellent electrocatalytic OER performance through an electrospinning‐calcination‐reduction process. Massive oxygen vacancies generated at the CeO_2_–CoO interface resulted in the formation of many low‐coordinated Co atoms, playing an essential role in acting as active sites and leading to the enhancement of OER activity.^[^ ^169^ ^]^ Moreover, Qiu et al. synthesized CeO_2_/Co_3_O_4_ nanotubes with the 2D–1D heterostructures and strongly coupled p–n heterointerfaces, which led to fast electron transfer (Figure 9d,e). Due to the abundant active sites, abundant oxygen vacancies, and enhanced conductivity, the catalyst showed excellent OER performance with an overpotential of 265 mV at 10 mA cm^−2^, a low Tafel slope of 68.1 mV dec^−1^, and enduring durability for OER.^[^ ^67^ ^]^

**Figure 10:**
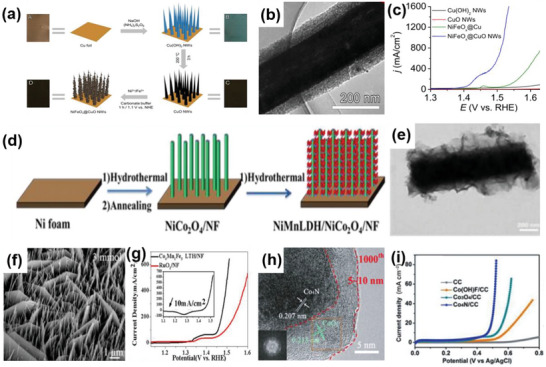
a) The synthesis process of Cu foil, Cu(OH)_2_ NWs/Cu, CuO NWs/Cu, and NiFeO*_x_* @CuO NWs/Cu. b) TEM image of NiFeO*_x_* @CuO NWs/Cu heterostructure. c) Polarization curves of NiFeO*_x_* @CuO NWs/Cu heterostructure. Reproduced with permission.^[^ ^69^ ^]^ Copyright 2018, Wiley‐VCH. d) The fabrication process of NiMn LDH/NiCo_2_O_4_ heterostructure. e) TEM image of the NiMn LDH/NiCo_2_O_4_. Reproduced with permission.^[^ ^70^ ^]^ Copyright 2018, Elsevier B.V. f) SEM image of Co_4_Mn_1_Fe_3_ LTH/NF. g) LSV curves of Co_4_Mn_1_Fe_3_ LTH/NF and the commercial catalyst. Reproduced with permission.^[^ ^71^ ^]^ Copyright 2019, Royal Society of Chemistry. h) HRTEM images of Co_4_N NW after 1000 CV cycles. i) Polarization curves of Co(OH)F NW, Co_3_O_4_ NW, and Co_4_N NW grown on carbon cloth. Reproduced with permission.^[^ ^72^ ^]^ Copyright 2015, Wiley‐VCH.

Another 2D–1D heterostructure that can be formed is the core–shell nanotubes. Wu et al. assembled Ni–Co hydroxide nanosheets onto nickel coated with CuO nanowires. They achieved this through a series of processes, including chemical oxidation, calcination, electro‐deposition, and the hydrothermal method, resulting in the formation of a core–shell nanoarray structure on the copper foam (Figure 9f,g). The CuO NWs provide a large surface area, while the Ni metal layer acts as a conductive component in terms of electrical conductivity. The resulting core–shell structure possessed a rough, porous surface that facilitated the exposure of active sites, gas release, and electron transfer. As a result, the NiCo/Ni/CuO/CF catalyst exhibits excellent electrocatalytic activity and durability, with a small overpotential of 240 mV at 10 mA cm^−2^ and a low Tafel slope of 37.9 mV dec^−1^.^[^ ^68^ ^]^ In another instance, Czioska et al. adopted a solution‐based method followed by a calcination process to fabricate a hierarchical 2D–1D NiFeO*_x_* nanosheets/CuO nanowires heterostructure on a planar copper foil as an electrocatalyst for OER (**Figure** **10**a–c). The high specific area contributed to the excellent catalytic performance, resulting in a small Tafel slope of 36 mV dec^−1^ and a low overpotential of 300 mV at 100 mA cm^−2^. This work proved that such a hierarchical heterostructure can significantly improve the catalytic efficiency of NiFeO*_x_*.^[^ ^69^ ^]^

Surface‐rich high‐valence metal oxides are beneficial for achieving a high‐efficiency catalyst for OER. Thus, Yang et al. constructed a hierarchical catalyst composed of 2D NiMn LDH nanosheets and 1D NiCo_2_O_4_ nanowires (Figure 10d). Figure 10e shows thin NiMn LDH nanosheets coated on NiCo_2_O_4_ nanowires. This work found that high‐valence Ni and Mn oxides greatly facilitate the charge transport between the electrode and electrolyte. In addition to the high valence, the synergistic effect, high electrical conductivity, and efficient active sites also play key roles in the oxygen generation process. As expected, NiMn LDH/NiCo_2_O_4_ can achieve a low overpotential of 310 mV at 10 mA cm^−2^, with a small Tafel slope of 99 mV dec^−1^. Additionally, it exhibited good stability for up to 8 h.^[^ ^70^ ^]^ Similarly, Guo et al. utilized a hydrothermal strategy to synthesize the novel CoMnFe layered triple hydroxide (LTH) arrays grown on NF, which possessed a unique 2D–1D nanosheet‐nanowire structure (Figure 10f,g). By rationally adjusting the incorporation of Fe, the distribution of active sites, electronic conductivity, and gas release channel can be properly optimized. Thus, the optimized Co_4_Mn_1_Fe_3_ LTH/NF can achieve a small overpotential of 226 mV at 100 mA cm^−2^, with a Tafel slope of 55 mV dec^−1^.^[^ ^71^ ^]^ Additionally, Chen et al. constructed Co_4_N porous nanowire arrays grown on carbon via a nitridation reaction (Figure 10h,i). During the OER process, the Co atoms on the surface of Co_4_N were partially oxidized into CoOOH, forming the active sites CoOOH/Co_4_N. At higher potential, the CoOOH/Co_4_N species was further oxidized to form the CoO_2_/Co_4_N complex species. Due to the high conductivity of metallic Co_4_N nanowires and the porous structure, the Co_4_N/CC catalyst exhibited good performance for OER, with an overpotential of 257 mV at 10 mA cm^−2^ and a Tafel slope of 44 mV dec^−1^ in an alkaline medium.^[^ ^72^ ^]^

#### 2.2.3 OER on 2D–2D Heterostructures

**Figure 11:**
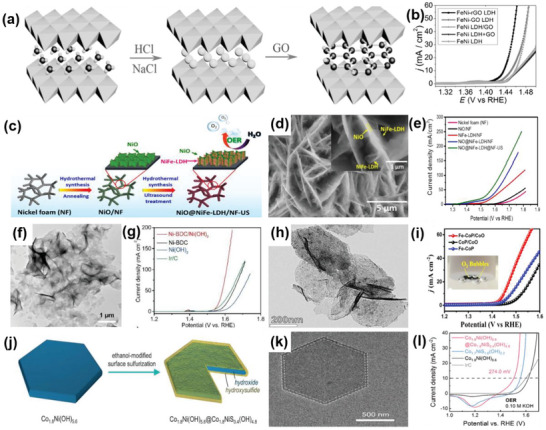
a) Fabrication procedure of FeNi–GO LDHs heterostructure. b) Polarization curves of FeNi–GO LDHs heterostructure and other samples. Reproduced with permission.^[^ ^73^ ^]^ Copyright 2014, Wiley‐VCH. c) Synthesis process of NiO@NiFe–LDH/NF heterostructure. d) SEM image of NiO@NiFe–LDH/NF. e) LSV curves of NiO@NiFe–LDH/NF and other samples. Reproduced with permission.^[^ ^74^ ^]^ Copyright 2019, American Chemical Society. f) TEM image of Ni–BDC/Ni(OH)_2_. g) LSV curves of Ni–BDC/Ni(OH)_2_ and other catalysts. Reproduced with permission.^[^ ^75^ ^]^ Copyright 2019, Royal Society of Chemistry. h) TEM image of Fe–CoP/CoO heterostructure. i) Polarization curves of Fe–CoP/CoO heterostructure and other samples. Reproduced with permission.^[^ ^76^ ^]^ Copyright 2018, Elsevier Ltd. j) Schematic illustration of the synthesis process of Co_1.8_Ni(OH)_5.6_@Co_1.8_NiS_0.4_(OH)_4.8_ heterostructure. k) TEM image of Co_1.8_Ni(OH)_5.6_@Co_1.8_NiS_0.4_(OH)_4.8_. l) LSV curves of Co_1.8_Ni(OH)_5.6_@Co_1.8_NiS_0.4_(OH)_4.8_ and other samples. Reproduced with permission.^[^ ^77^ ^]^ Copyright 2018, Wiley‐VCH.

2D–2D heterostructures can significantly enhance the regulation of electron density and energy bands, making it easier to adsorb and desorb the intermediate products of OER. The rapid transmission of electrons within the plane improves the catalytic activity of the heterostructure. The 2D materials possess good mechanical strength provided by covalent bonding within the plane and weak bonding forces formed by vdW interactions or electrostatic interactions between the layers. Thus, it can be easily peeled off and self‐assembled with other 2D materials to form heterostructures. For example, Long et al. combined layered FeNi double hydroxide with graphene to produce a FeNi–GO LDH catalyst (**Figure** **11**a,b). The synergistic effect between FeNi LDH and graphene led to an enhanced catalytic performance for OER, with a Tafel slope close to that of the Ir/C catalyst, which is 40 mV dec^−1^.^[^ ^73^ ^]^ Another example is provided by Sirisomboonchai et al., who adopted a two‐step hydrothermal method to synthesize 2D–2D NiO microflake@NiFe–LDH nanosheet core–shell arrays on NF (Figure 11c–e). The required overpotential for OER to sustain a current density of 10 mA cm^−2^ was reduced from 265 to 210 mV after ultrasonic treatment.^[^ ^74^ ^]^ Furthermore, Zhu et al. synthesized 2D–2D Ni–BDC/Ni(OH)_2_ heterostructure nanosheets through a sonication‐assisted solution process (Figure 11f,g). The coupling operation can effectively modify the electronic structure of Ni atoms in the Ni(OH)_2_ component and lead to the production of Ni cations with higher oxidation states, thereby enhancing the performance of OER. As expected, the Ni–BDC/Ni(OH)_2_ heterostructure exhibited an overpotential of 320 mV at 10 mA cm^−2^, a Tafel slope of 41 mV dec^−1^, and strong durability, with the activity maintained for 20 h.^[^ ^75^ ^]^

MOFs are formed through coordination bonds between organic ligands and metal atom nodes. The unsaturated coordination sites on the metal atom surface can improve the catalytic efficiency of OER. Nevertheless, the active metal centers in MOFs are blocked by organic ligands, resulting in a decrease in their catalytic efficiency. When MOFs are transformed into 2D nanosheet structures, a large number of active sites are exposed, leading to high catalytic efficiency. 2D MOFs are generally considered valuable electrocatalysts for OER due to their rapid mass transfer, large number of unsaturated metal atoms, and high conductivity. For example, Duan et al. adopted a facile one‐step chemical bath deposition approach to grow 2D MOF structures in situ on various supports. The highly exposed metallic NiFe center makes the MOF nanosheets highly hydrophilic, facilitating water adsorption on the electrode surface and promoting the kinetics of water dissociation. Moreover, its substantial metal molecular active sites increase electrical conductivity, and the combination of hierarchical porosity also contributes to enhancing catalytic properties. As a result, the 2D NiFe–MOF exhibited outstanding performance, including an overpotential of 240 mV at 10 mA cm^−2^, a low Tafel slope of 34 mV dec^−1^, and a TOF of 3.8 s^−1^ at 400 mV for OER.^[^ ^170^ ^]^

Apart from the NiFe compound, FeCo and CoNi compounds also show good performance toward OER. For example, Hu et al. used an instantaneous method to synthesize FeCo–LDH/CoO nanosheets in a 2D–2D structure (Figure 11h,i). Owing to the strong coupling effect, the catalyst exhibited superior electrocatalytic activity (η_10_ = 219 mV).^[^ ^76^ ^]^ Moreover, Wang et al. synthesized a core–shell heterostructure of 2D–2D Co_1.8_Ni(OH)_5.6_@Co_1.8_NiS_0.4_(OH)_4.8_ using a method of ethanol‐modified surface sulfurization (Figure 11j–l). The catalyst exhibited exceptional OER performance with an overpotential of 274 mV at 10 mA cm^−2^ and a Tafel slope of 45 mV dec^−1^ in alkaline conditions, which can be attributed to its rapid kinetics.^[^ ^77^ ^]^

Layered MoS_2_ is an excellent HER electrocatalyst, but it exhibits poor OER activity. Therefore, enhancing the catalytic performance for OER is a necessary step toward achieving overall water splitting. For instance, Xue et al. conducted systematic theoretical research on the electrocatalytic activity of the 2D–2D MoS_2_/g–C_3_N_4_ heterostructure for OER. The MoS_2_/g–C_3_N_4_ heterostructure can effectively modify the electronic properties of the MoS_2_ phase by reducing its bandgap, resulting in a nearly 69% reduction in overpotential. This work provides theoretical support for the utilization of MoS_2_ heterostructured material as an OER catalyst.^[^ ^78^ ^]^

#### 2.2.4 OER on 2D–3D Heterostructures

**Figure 12:**
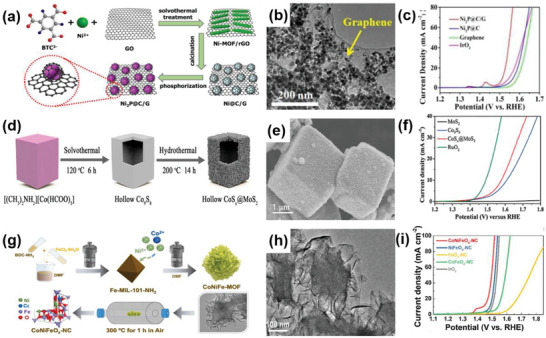
a) Scheme for the formation of Ni_2_P@C/G heterostructure. b) TEM image of Ni_2_P@C/G. c) LSV curves of Ni_2_P@C/G and other samples. Reproduced with permission.^[^ ^79^ ^]^ Copyright 2017, Royal Society of Chemistry. d) Scheme for the fabrication procedure of CoS*_x_* @MoS_2_ heterostructure. e) FESEM image of CoS*_x_* @MoS_2_ microcubes. f) Polarization curves of CoS*_x_* @MoS_2_ and other samples. Reproduced with permission.^[^ ^80^ ^]^ Copyright 2018, American Chemical Society. g) Scheme for the formation of CoNiFeO*_x_*–NC heterostructure. h) TEM image of CoNiFeO*_x_*–NC. i) LSV curves of CoNiFeO*_x_*–NC and other samples. Reproduced with permission.^[^ ^81^ ^]^ Copyright 2021, Elsevier B.V.

The heterostructures formed by the combination of 3D structure and 2D nanosheets possess a large contact area, which allows for better control over the properties of the 2D material and improves its catalytic activity. Additionally, many 2D–3D heterostructures exhibit various types of banding that can enhance the hydrophilicity of the material and facilitate water decomposition. For example, Wang et al. constructed 3D Ni_2_P nanocrystals supported on 2D graphene through solvothermal treatment, calcination, and phosphorization (**Figure** **12**a). Figure 12b shows that monodispersed Ni_2_P@C NPs were distributed on the graphene surface. Due to the introduction of graphene, the catalytic properties of Ni_2_P@C/G were significantly improved, including increased exposure of active sites and accelerated charge transfer. Based on these upgraded properties, the OER performance was correspondingly enhanced (Figure 12c), with a low overpotential of 285 mV at 10 mA cm^−2^, a Tafel slope of 44 mV dec^−1^, and remarkable durability of up to 10 h.^[^ ^79^ ^]^ In another example, Yang et al. successfully grew massive 2D MoS_2_ nanosheets vertically and uniformly on the surface of the 3D CoS_2_ hollow structure through solvothermal and hydrothermal methods (Figure 12d–f). The CoS_2_@MoS_2_ electrocatalyst showed favorable properties for the catalytic process, including a large effective surface area, high electrical conductivity, and long‐term stability. Especially, the synergistic effect of the CoS_2_@MoS_2_ heterostructure played a key role in improving the catalytic activity.^[^ ^80^ ^]^

The catalytic activity can be improved by the core–shell structure formed by the 2D–3D heterostructure. For instance, Chen et al. utilized the ion‐exchange‐based method to synthesize a hierarchically structured Co–Ni–Fe spinel oxide‐carbonitride, where a 2D ternary metal MOF shell layer encapsulated 3D octahedral MOF crystals (Figure 12g–i). This core–shell structure provided a favorable electronic environment for OER intermediates. Additionally, the Ni–Co coordinated O_h_ sites, serving as the main active sites, can tune the binding strength of O species via electron transfer, which was beneficial for lowering the energy barriers toward OER. Therefore, the CoNiFeO*_x_*–NC electrocatalyst exhibited excellent catalytic performance, with an overpotential of 265 mV at 50 mA cm^−2^ and a Tafel slope of 64.1 mV dec^−1^.^[^ ^81^ ^]^ Moreover, Tong et al. reported a self‐supported heterostructure that consisted of Co(OH)_2_ nanosheets decorated with Ag NPs and supported on N‐doped carbon nanoflake arrays on carbon cloth. This Ag/Co(OH)_2_@NC/CC heterostructure electrocatalyst demonstrated excellent performance, mainly attributed to the 3D structure consisting of Co(OH)_2_ and NC with rich ion transport pathways, the interconnected Co(OH)_2_ nanosheets with a large surface area, and NC nanoflake arrays with high electrical conductivity.^[^ ^82^ ^]^

**Figure 13:**
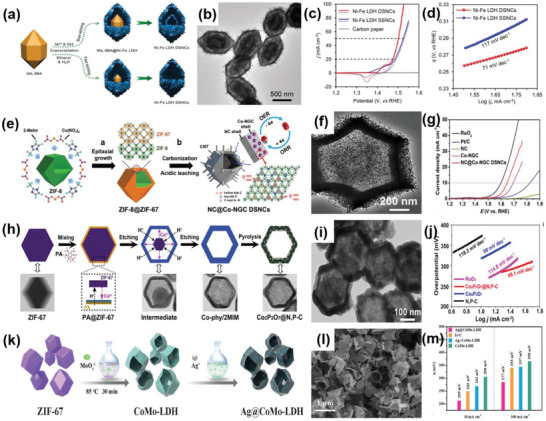
a) Schematic illustration of the formation of Ni–Fe LDH nanocage heterostructure. b) SEM image of Ni–Fe LDH DSNCs. c,d) CV curves and Tafel slopes of Ni–Fe LDH DSNCs and other samples. Reproduced with permission.^[^ ^83^ ^]^ Copyright 2020, Wiley‐VCH. e) Scheme for the formation of NC@Co–NGC DSNCs heterostructure. f) TEM image of NC@Co–NGC DSNCs heterostructure. g) Polarization curves of NC@Co–NGC DSNCs heterostructure and other catalysts. Reproduced with permission.^[^ ^84^ ^]^ Copyright 2017, Wiley‐VCH. h) Schematic illustration of the fabrication procedure of the Co_2_P_2_O_7_@N,P‐C nanocages heterostructure. i) TEM image of the Co_2_P_2_O_7_@N,P‐C heterostructure. j) Tafel plots of the Co_2_P_2_O_7_@N,P‐C heterostructure, and other samples. Reproduced with permission.^[^ ^85^ ^]^ Copyright 2019, Elsevier B.V. k) Schematic illustration of the preparation of Ag@CoMo–LDH heterostructure. l) SEM image of Ag@CoMo–LDH. m) Required overpotentials at certain current densities of Ag@CoMo–LDH and other catalysts. Reproduced with permission.^[^ ^86^ ^]^ Copyright 2022 Elsevier Inc.

2D–3D heterostructure can also take the form of hollow structures, especially with nanocage morphology, resulting in a large specific surface area that can expose more active sites. The orderly arrangement of the 2D planes serves as a channel for transporting reaction substances, while also maintaining the coupling effect between planes to enhance the adsorption and desorption of intermediate products. For example, in **Figure** **13**a–d, Zhang et al. obtained NiFe–LDH double‐shelled nanocages (DSNCs) through simultaneous etching and coprecipitation reactions. The number of shells in NiFe–LDH nanocages can be easily controlled by adjusting the volume ratio of ethanol to water. The catalyst showed high activity toward OER, with an overpotential of 246 mV at 20 mA cm^−2^, attributed to the small charge transfer resistance.^[^ ^83^ ^]^ Similarly, Liu et al. drew inspiration from the desired architecture of hollow structures. By creating a hollow chamber within the catalyst, this structure introduced an extra triple‐phase interface for OER/ORR and also prevented the aggregation phenomenon. Liu's work focused on synthesizing DSNCs, where the internal shell consisted of N‐doped carbon, and the outer shell was Co‐decorated N‐doped graphitic carbon (Figure 13e–g). These nanocages greatly combined the high catalytic activity of the outer shell with the rapid diffusion kinetics of the inner shell, achieving high‐performance bifunctional catalysis for OER/ORR, surpassing the performance of Pt and RuO_2_ electrocatalysts.^[^ ^84^ ^]^

Furthermore, Liang et al. reported Co_2_P_2_O_7_@N,P co‐doped carbon nanocages with the Mott–Schottky heterostructure (Figure 13h–j). The difference in the Fermi level played a critical role in facilitating the flow of valence electrons at the interface of the heterostructure. The N, P codoping carbon layer primarily modulated the Co center's e_g_ orbital occupation of Co_2_P_2_O_7_. The exceptional OER activity and durability of the electrocatalyst obtained in this study further highlighted the importance of modulating the electronic structure to achieve high efficiency in OER.^[^ ^85^ ^]^ Also, Zhang et al. employed a template method and a spontaneous strategy to synthesize a metal‐support nanocage heterostructure consisting of Ag NPs and CoMo–LDH (Figure 13k–m). The rapid transfer of electrons and mass can optimize the activity of initial sites. Additionally, the formation of efficient and stable heterointerfaces between Ag and CoMo–LDH contributed to the appearance of new active sites. Consequently, Ag@CoMo–LDH exhibited remarkable OER performance, with an overpotential of 205 mV at 10 mA cm^−2^ and a Tafel slope of 45.7 mV dec^−1^. In addition to the activity, Ag@CoMo–LDH also showed long‐term stability. This work demonstrated that constructing a new LDH‐based OER catalyst can effectively improve catalytic performance.^[^ ^86^ ^]^

### 2.3 OWS

Catalysts for OER and HER may require different types of electrolytes to maintain optimal catalytic activity and stability. However, the simultaneous use of two materials as electrodes in electrolyzed water is likely to result in a decrease in catalytic efficiency. In addition, different techniques for preparing electrode materials can complicate the production and application processes. Therefore, applying materials with both OER and HER catalytic activity as cathode and anode catalysts in electrolyzed water holds great application prospects. However, finding a material perfectly matched with outstanding catalytic efficiency is difficult. Heterostructures can combine the advantages of two different types of catalytic materials to achieve OER and HER catalysis simultaneously. The catalytic efficiency of a heterostructure is even better than what is achieved by the corresponding single material alone. This section summarizes the cases of 2D materials combined with 0D, 1D, 2D, or 3D materials to form heterostructures for OWS, which clearly reflect the advantages of heterostructures with different structures.

#### 2.3.1 OWS on 2D–0D Heterostructures

**Figure 14:**
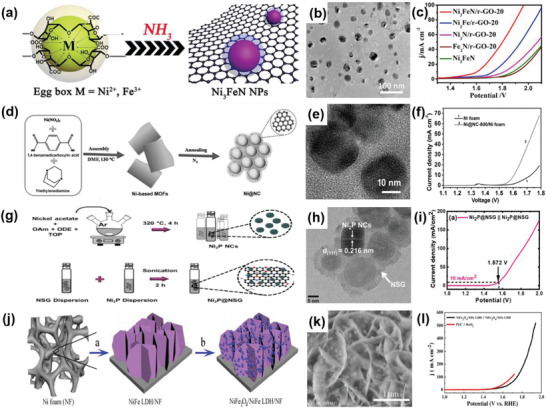
a) Illustration for the synthesis of Ni_3_FeN/r‐GO heterostructure. b) TEM image of Ni_3_FeN/r‐GO‐20. c) OWS performance of Ni_3_FeN/r‐GO‐20 and other samples. Reproduced with permission.^[^ ^87^ ^]^ Copyright 2018, American Chemical Society. d) The preparation workflow of Ni@NC heterostructure. e) TEM image of the Ni@NC. f) LSV of Ni@NC for OWS. Reproduced with permission.^[^ ^89^ ^]^ Copyright 2017, Wiley‐VCH. g) Schematic illustration of the fabrication processes of Ni_2_P@NSG heterostructure. h) HRTEM image of Ni_2_P@NSG. i) Polarization curve of Ni_2_P@NSG for OWS. Reproduced with permission.^[^ ^90^ ^]^ Copyright 2021, American Chemical Society. j) Preparation process of NiFe_2_O_4_/NiFe LDH heterostructure array. k) High‐magnification FESEM image of NiFe_2_O_4_/NiFe LDH. l) LSV curves of NiFe_2_O_4_/NiFe LDH and commercial catalysts for OWS. Reproduced with permission.^[^ ^91^ ^]^ Copyright 2018, American Chemical Society.

The bi‐transition metal center and N coordination in Ni_3_FeN enable the material to possess both OER and HER catalytic activity, but its conductivity is poor. The formation of a 2D–0D heterostructure with graphene can improve the overall conductivity and catalytic efficiency. For example, Gu et al. successfully obtained Ni_3_FeN NPs/rGO aerogel heterostructure electrocatalysts through an ion exchange and a one‐step nitrogenization process (**Figure** **14**a). It can be observed that the carbon shell is coated on the Ni_3_FeN NPs from Figure 14b. In addition to the advantages of having pores, the hydrogel structure allowed the catalyst to be directly utilized in the electrode, resulting in good stability during the reaction. When the current density reached 10 mA cm^−2^, Ni_3_FeN/rGO only needed an overpotential of 270 mV for OER. It also demonstrated excellent HER catalytic activity with an overpotential of 94 mV at 10 mA cm^−2^. When serving as both the cathode and the anode for OWS (Figure 14c), the Ni_3_FeN/rGO catalyst achieved a current density of 10 mA cm^−2^ at 1.60 V, with a durability of 100 h. DFT calculation results revealed that this good performance resulted from the alteration in the surface electronic structure caused by the contact of Ni_3_FeN NPs with rGO.^[^ ^87^ ^]^ Wang et al. treated freeze‐dried powder of GO and CoCl_2_ with phosphate at low temperatures to reduce GO and synthesize CoP_2_/rGO materials. 0D CoP_2_ NPs were grown uniformly on the 2D GO nanosheets. The Co in CoP acted as a hydride‐acceptor, and the P in CoP acted as a proton acceptor center, promoting the generation of co‐hydrides through electrochemical desorption. As a result, this heterostructure exhibited excellent catalytic performance. For OER catalytic activity, the CoP_2_/rGO required an overpotential of 300 and 330 mV to offer a current density of 10 and 20 mA cm^−2^, respectively, slightly higher than that of RuO_2_ (270, 300 mV). Additionally, the catalyst merely required a cell voltage of 1.56 V at 10 mA cm^−2^ for OWS.^[^ ^88^ ^]^

In addition, the utilization of 2D material encapsulating NPs to form a 2D–0D heterostructure is also an effective approach to enhance electrocatalytic activity. For instance, Xu et al. successfully fabricated a Ni@NC heterostructure using an annealing approach (Figure 14d–f). The structure of the obtained product was 0D Ni NP cores coated with several 2D graphene shells. Due to the synergistic effect, the catalyst exhibited good catalytic activity and stability for both the HER and OER under alkaline conditions.^[^ ^89^ ^]^ Furthermore, as shown in Figure 14g, Suryawanshi et al. established a heterostructure consisting of 0D NiP NPs and 2D N‐ and S‐doped graphene nanosheets (NSG). Specifically, NiP NPs were encapsulated in NSG (Figure 14h). The strong interaction between the NiP cores and NSG shells through the vdW force was conducive to enlarging the active surface area and increasing the exposure of active sites. As a result, NiP@NSG catalysts displayed excellent performance toward OWS. An alkaline electrolyzer using them as electrocatalysts for HER and OER only required 1.572 V at 10 mA cm^−2^ to function (Figure 14i).^[^ ^90^ ^]^

As reported, NiFe_2_O_4_ can be suitable for serving as an electrocatalyst for OWS due to its good conductivity, rich surface redox, abundance in the earth's crust, and environmental friendliness. However, NiFe_2_O_4_‐based catalysts still possess some shortcomings, including low reactivity and a limited number of active sites, thus limiting the enhancement of catalysis properties. As for NiFe–LDH, the unique structure enables it to couple with other materials to enhance OWS activity. Therefore, in order to improve OWS catalytic performance, Wu et al. fabricated the NiFe_2_O_4_/NiFe heterostructure, which combined 0D NiFe_2_O_4_ NPs and 2D LDH nanosheets (Figure 14j). NiFe_2_O_4_ NPs are uniformly attached to the surfaces of the LDH nanosheet (Figure 14k). After a series of instrumental tests, the prepared heterostructure catalyst can achieve a current density of 100 mA cm^−2^ at a low overpotential of 213 mV for OER and require a small overpotential of 101 mV at 10 mA cm^−2^ for HER. Furthermore, when this catalyst was used as both the anode and the cathode in a two‐electrode electrolyzer, it showed a low voltage of 1.535 V at 10 mA cm^−2^ (Figure 14l). These outstanding catalytic properties were mainly attributed to the strong coupling effect and the presence of abundant active sites.^[^ ^91^ ^]^ Moreover, MoS_2_ has a high H‐adsorption capacity, while Ni_3_S_2_ possesses a high HO‐adsorption capacity. Thus, Zhang et al. utilized this principle to prepare a bifunctional electrocatalyst with a 2D–0D MoS_2_/Ni_3_S_2_ heterostructure. The outer MoS_2_ nanosheets were fixed on the surface of the inner Ni_3_S_2_ NPs. This structure led to the formation of abundant interfaces, which was beneficial for improving catalytic activity. Consequently, the catalyst showed comparatively excellent catalytic activity for OWS with a current density of 10 mA cm^−2^ at 1.56 V.^[^ ^92^ ^]^

#### 2.3.2 OWS on 2D–1D Heterostructures

**Figure 15:**
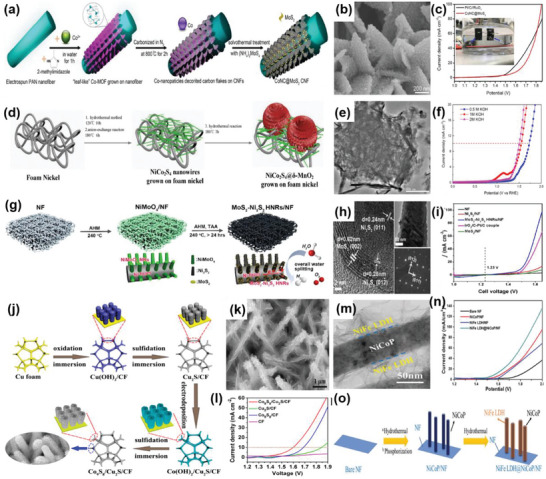
a) Illustration of the fabrication process of CoNC@MoS_2_/CNF heterostructure. b) SEM image of CoNC@MoS_2_/CNFs. c) LSV curves of CoNC@MoS_2_/CNFs for OWS. Reproduced with permission.^[^ ^93^ ^]^ Copyright 2017, Royal Society of Chemistry. d) Synthesis illustration of a NiCo_2_S_4_@δ–MnO_2_ heterostructure. e) TEM image of NiCo_2_S_4_@δ–MnO_2_. f) Polarization curves of NiCo_2_S_4_@δ–MnO_2_ for OWS. Reproduced with permission.^[^ ^94^ ^]^ Copyright 2019, Elsevier Ltd. g) Fabrication process of MoS_2_−Ni_3_S_2_ HNRs/NF heterostructure. h) TEM images of the MoS_2_–Ni_3_S_2_ HNRs/NF heterostructure. i) Polarization curves of the MoS_2_–Ni_3_S_2_ HNRs/NF heterostructure for OWS. Reproduced with permission.^[^ ^95^ ^]^ Copyright 2017, American Chemical Society. j) Synthesis Procedure for the Co_9_S_8_/Cu_2_S/CF heterostructure. k) SEM image of Co_9_S_8_/Cu_2_S/CF. l) LSV curves of Co_9_S_8_/Cu_2_S/CF for OWS. Reproduced with permission.^[^ ^96^ ^]^ Copyright 2021, American Chemical Society. m) TEM image of a NiFe–LDH@NiCoP heterostructure. n) Polarization curves of NiFe–LDH@NiCoP heterostructure for OWS. o) Schematic representation of the synthesis of NiFe LDH@NiCoP/NF heterostructure. Reproduced with permission.^[^ ^97^ ^]^ Copyright 2018, Wiley‐VCH.

In nature, leaves have a natural 2D–1D structure, and the veins within the leaf efficiently transport water. When comparing water molecules to electrons, embedding a 2D plane within a 1D fiber allows for rapid electron conduction, thereby speeding up the reaction rate. For example, Wang et al. synthesized CoNC/electrospun carbon nanofibers (CNFs) and then used a solvothermal method to graft MoS_2_ nanosheets onto them (**Figure** **15**a). As observed in Figure 15b, the MoS_2_ nanosheets exhibit vertically exposed edges and are embedded within a porous CoNC structure. The 2D–1D CoNC@MoS_2_–CNF heterostructures possessed favorable flexibility and high electrical conductivity, which were conducive to improving the catalytic performance. As expected, this catalyst displayed high catalytic activity for HER (overpotential of 143 mV, Tafel slop of 68 mV dec^−1^) and OER (overpotential of 350 mV, Tafel slop of 51.9 mV dec^−1^). The catalyst required an overpotential of 1.62 V to reach 10 mA cm^−2^ for OWS. Its catalytic activity was even superior to that of the Pt/C–RuO_2_ electrode with a large current density (Figure 15c).^[^ ^93^ ^]^

For 2D–1D heterostructures, the more common structures involve 1D nanomaterials embedded within a 2D plane or vertically arranged on the surface of 2D materials. For example, Zhang et al. reported a spherical cactus‐like structure, where 1D NiCo_2_S_4_ nanowires were interspersed within 2D layered δ‐MnO_2_ (Figure 15d–f). Due to rapid electron transfer and the synergistic effect, the catalyst obtained exhibited good OWS catalytic performance, with an overpotential of 1.55 V at 10 mA cm^−2^ in 1 m KOH.^[^ ^94^ ^]^ Besides, Yang et al. prepared MoS_2_ nanosheets on Ni_3_S_2_ nanorods (Figure 15g–i). This heterostructure possessed a fast charge transport ability and a high exposure of active heterointerfaces, which were beneficial for catalytic activity. As expected, it only involved a cell voltage of 1.50 V to drive a current density of 10 mA cm^−2^.^[^ ^95^ ^]^ Similarly, Zang et al. employed impregnation and electrodeposition techniques to prepare 2D Co_9_S_8_ nanosheets coupled with 1D CuS nanorods, which functioned as an electrocatalyst for OWS (Figure 15j–l). The interfacial interaction increased electron interaction and accelerated charge transfer, leading to excellent catalytic performance with a low overpotential of 195 mV at 10 mA cm^−2^ for OER and 165 mV for HER at the same current density.^[^ ^96^ ^]^

Growing heterostructured nanowires on a substrate is a good strategy for preparing an efficient OWS catalyst, which can synergistically utilize the HER and OER efficiencies of the two materials to exhibit excellent OWS efficiency. Meanwhile, the use of adhesives has been eliminated, resulting in a significant improvement in electrical conductivity. For example, Zhang et al. synthesized 2D–1D NiFe–LDH@NiCoP core@shell nanowires on the NF through a low‐temperature hydrothermal‐phosphorization‐hydrothermal process (Figure 15o). The core–shell structure and the interfaces between the nanowires and nanosheets can be observed in Figure 15m. The strong electronic interaction helped to facilitate charge transfer and enhance reaction kinetics. As a result, the bifunctional electrocatalysts only required a cell voltage of 1.57 V at 10 mA cm^−2^ for OWS (Figure 15n).^[^ ^97^ ^]^

#### 2.3.3 OWS on 2D–2D Heterostructures

2D materials‐based vdW heterostructures have shown promising applications for OWS due to their ease of fabrication. However, electron transport can be suppressed across the vdW gap, and the weak binding force can result in unstable electrocatalytic performance. To address this issue, Jiang et al. prepared 2D–2D CoNC@Co_2_N heterostructures, where Co_2_N was a non‐vdW material with a zero bandgap. The introduction of the 2D non‐vdW material, Co_2_N, and the strong connection between different materials led to improved performance. The CoNC@Co_2_N catalyst showed a low OWS overpotential of 1.52 V at 10 mA cm^−2^ in 1 m KOH and demonstrated excellent cycling stability. After 280 h, there was only a 20% decay in current density at 10 mA cm^−2^.^[^ ^171^ ^]^

**Figure 16:**
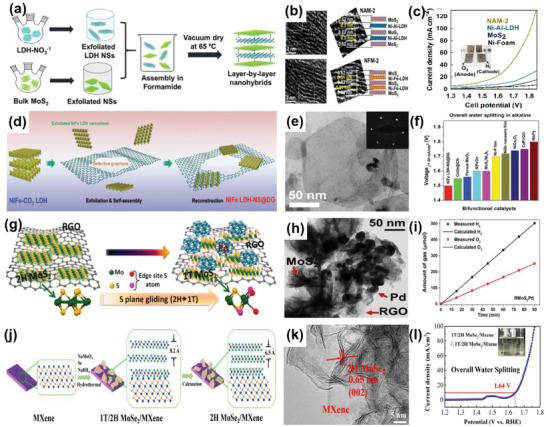
a) Schematic illustration of the fabrication process of LDH and MoS_2_. b) HRTEM images of constructed LDH/MoS_2_ heterostructure. c) LSV plots of constructed LDH/MoS_2_ heterostructure for OWS. Reproduced with permission.^[^ ^98^ ^]^ Copyright 2018, American Chemical Society. d) The fabrication process and e) TEM image of NiFe LDH–NS@DG heterostructure. f) Comparison of the required voltage for the displayed bifunctional catalysts. Reproduced with permission.^[^ ^99^ ^]^ Copyright 2017, Wiley‐VCH. g) Structural transformation of 2H–MoS_2_ to form 1T–MoS_2_/rGO/Pd heterostructure. h) TEM image of MoS_2_/rGO/Pd. i) The amount of H_2_ and O_2_ evolved of MoS_2_/rGO/Pd for OWS. Reproduced with permission.^[^ ^100^ ^]^ Copyright 2019, American Chemical Society. j) Schematic of the preparation of 2H MoSe_2_/MXene and 1T/2H MoSe_2_/Mxene heterostructure. k) TEM images of 2H MoSe_2_/MXene heterostructure. l) LSV curve of 2H MoSe_2_/MXene heterostructure for OWS. Reproduced with permission.^[^ ^101^ ^]^ Copyright 2019, Elsevier Ltd.

Moreover, LDH‐based vdW heterostructures also serve as excellent electrocatalysts for OWS by the formation of 2D–2D heterostructures with other 2D materials. For instance, Islam et al. synthesized strongly coupled heterostructures of LDH–TMD via the self‐assembly of cationic LDH and anionic TMD nanosheets (**Figure** **16**a–c). After interstratifying with MoS_2_ nanosheets, the chemical stability of LDH in an acidic solution was remarkably improved, which was beneficial to the catalytic performance. As expected, the heterostructure exhibited a low cell potential of 1.45 V at a current density of 10 mA cm^−2^ when using 1.0 m KOH as the electrolyte.^[^ ^98^ ^]^ Likewise, Jia et al. synthesized a 2D–2D NiFe LDH–NS@DG catalyst through electrostatic interaction, in which positively charged NiFe LDH nanosheets and negatively charged defective graphene were stacked together (Figure 16d,e). The DFT calculation indicated that the high electron density on DG facilitated HER, while the high density of holes on NiFe LDH–NS improved OER performance. As a result, the catalyst exhibited excellent electrocatalytic activity for HER (with an overpotential of 115 mV at 20 mA cm^−2^) and OER (with an overpotential of 0.21 V at 10 mA cm^−2^). For OWS, it only needed a voltage of 1.5 V to drive a current density of 20 mA cm^−2^. It can also be observed that it had excellent catalytic activity when comparing its performance with other OWS catalytic materials (Figure 16f).^[^ ^99^ ^]^

The 1T phase TMDs have substantial catalytic sites and high conductivity. When formed into a heterostructure with other 2D materials, they can be used as dual‐functional catalysts and possess excellent catalytic efficiency. For example, Pandey et al. prepared a ternary heterostructured material (RMoS_2_Pd) where Pd NPs were anchored onto the rGO/MoS_2_ layered structures (Figure 16g–i). When MoS_2_ transformed from the 2H phase to the 1T phase, it caused the transversal displacement of the S atom and created an S‐vacancy, leading to excellent catalytic activity. Thus, the rGO/MoS_2_/Pd catalyst showed low overpotentials (ŋ_10_ = 245 mV for OER and ŋ_10_ = 86 mV for HER), small Tafel slopes (42 mV dec^−1^ for OER and 35.9 mV dec^−1^ for HER), and low charge transfer resistance.^[^ ^100^ ^]^ Similarly, Li et al. synthesized 1T/2H MoSe_2_/MXene heterostructures through a simple one‐step hydrothermal method at 220 °C (Figure 16j–l). When annealed at 600 °C, the MoSe_2_ transformed to the 2H phase, and MXene could prevent the aggregation of MoSe_2_. This heterostructure possessed high conductivity provided by MXene and had massive active sites, leading to the enhancement of catalytic performance. Therefore, for OWS, the heterostructure electrocatalyst only required 1.64 V to attain a current density of 10 mA cm^−2^. The content of the 1T phase remained about 18% even after 50 h of overall water splitting at 1.64 V.^[^ ^101^ ^]^

**Figure 17:**
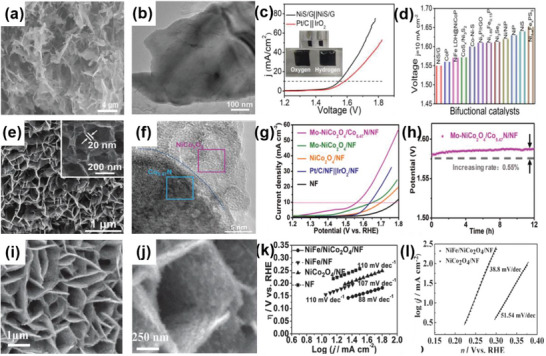
a,b) SEM and TEM images of NiS/G heterostructure. c) LSV curve of NiS/G for OWS. d) Comparison of overpotentials between NiS/G and other catalysts toward OWS. Reproduced with permission.^[^ ^102^ ^]^ Copyright 2019, Elsevier B.V. e,f) SEM and HRTEM images of Mo–NiCo_2_O_4_/Co_5.47_N/NF. g) Polarization curves of Mo–NiCo_2_O_4_/Co_5.47_N/NF and other samples for OWS. h) Stability of Mo–NiCo_2_O_4_/Co_5.47_N/NF for OWS. Reproduced with permission.^[^ ^103^ ^]^ Copyright 2020, Wiley‐VCH. i,j) SEM images of NiFe/NiCo_2_O_4_ /NF heterostructure. k) Tafel plots of NiFe/NiCo_2_O_4_ /NF heterostructure and other samples for HER. l) Tafel plots of NiFe/NiCo_2_O_4_/NF heterostructure and other samples for OER. Reproduced with permission.^[^ ^104^ ^]^ Copyright 2016, Wiley‐VCH.

It is known that NiS is a promising catalyst for water splitting, but it suffers from poor stability and high overpotential in alkaline media, thus limiting the electrocatalytic performance. However, when combined with graphene, the electrocatalytic activity and stability of NiS can be greatly improved. Zhang et al. conducted a study where they combined 2D graphene with 2D NiS nanosheets, resulting in a high exposure of surface active sites (**Figure** **17**a,b). The strong coupling not only enhanced conductivity but also enriched the surface with active sites, further improving the electrocatalytic activity. As expected, the catalyst exhibited excellent catalytic performance, as demonstrated by a low cell voltage of 1.54 V at 10 mA cm^−2^ in an alkaline solution for OWS, surpassing the performance of both Pt/C and IrO_2_ electrodes. Comparative analysis with other bifunctional catalytic materials objectively proved that G‐NiS exhibited outstanding catalytic activity (Figure 17c,d).^[^ ^102^ ^]^

Another smart catalyst design is to grow a proper structure onto a conductive porous support, such as NF, which can provide maximal exposure of active sites and rapid mass transport ability. For example, Liu et al. introduced a non‐3d high‐valence metal into a heterostructure electrocatalyst with a controlled microstructure. They used thermal treatment to obtain a Mo‐doped porous NiCo_2_O_4_/Co_5.47_N nanosheet array coated on NF (Figure 17e–h). The authors believed that the non‐3d high‐valence metal‐doped heterostructure conferred optimized electronic structure and improved catalytic activity upon the catalyst. Thus, this heterostructure showed excellent catalytic properties, including an overpotential of 310 mV at 50 mA cm^−2^ for OER and 170 mV for HER. For OWS, it required a low voltage of 1.56 V at 10 mA cm^−2^.^[^ ^103^ ^]^ Similarly, Xiao et al. prepared a 2D–2D heterostructure consisting of NiCo_2_O_4_ nanoflakes, NiFe nanosheets, and NF using a hydrothermal method and electrodeposition (Figure 17i–l). The electrode consisted of three grades of porous structures, and this structure possessed an abundance of active sites, which were conducive to promoting catalytic performance. As a result, the heterostructure required only a potential of 1.67 V to achieve a current density of 10 mA cm^−2^ for OWS.^[^ ^104^ ^]^

#### 2.3.4 OWS on 2D–3D Heterostructures

**Figure 18:**
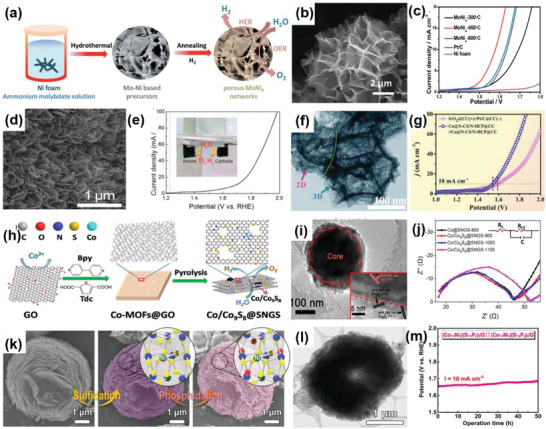
a) Illustration of the formation of MoNi_4_ networks. b) SEM image of porous MoNi_4_/NF. c) Polarization curves of porous MoNi_4_/NF at different temperatures for OWS. Reproduced with permission.^[^ ^105^ ^]^ Copyright 2017, Royal Society of Chemistry. d) SEM image of CoMnCH/NF. e) LSV of CoMnCH/NF for OWS. Reproduced with permission.^[^ ^106^ ^]^ Copyright 2017, American Chemical Society. f) TEM image of Co@N–CS/N–HCP heterostructure. g) LSV curves of Co@N–CS/N–HCP@CC and other samples for OWS. Reproduced with permission.^[^ ^107^ ^]^ Copyright 2019, Wiley‐VCH. h) Schematic illustration of the formation process of Co/Co_9_S_8_@SNGS heterostructure. i) HRTEM image of Co/Co_9_S_8_@SNGS. j) EIS of Co/Co_9_S_8_@SNGS heterostructures. Reproduced with permission.^[^ ^108^ ^]^ Copyright 2016, Elsevier Ltd. k) Schematic diagram of the fabrication process and SEM images of (Co_1−_ *_x_* Ni*_x_*)(OH)_2_/G, (Co_1−_ *_x_* Ni*_x_*)S_2_/G, and (Co_1−_ *_x_* Ni*_x_*)(S_1−_ *_y_* P*_y_*)_2_/G heterostructure. l) TEM image of (Co_1−_ *_x_* Ni*_x_*)(S_1−_ *_y_* P*_y_*)_2_/G. m) Chronopotentiometric curve of (Co_1−_ *_x_* Ni*_x_*)(S_1−_ *_y_* P*_y_*)_2_/G for OWS. Reproduced with permission.^[^ ^109^ ^]^ Copyright 2018, Wiley‐VCH.

Porous structures can accelerate the adsorption and desorption of intermediate substances, thereby enhancing the catalytic efficiency of OWS. NF, as a porous template, can be utilized in the synthesis of catalysts for OWS. For instance, Jin et al. grew MoNi_4_ networks on porous NF via a hydrothermal process followed by an annealing method in hydrogen (**Figure** **18**a–c). The porous structure and NiOOH species grown on the surface of MoNi_4_ networks contributed to the high performance for OER, including a potential of about 1.51 V at 10 mA cm^−2^ and a Tafel slope of 79 mV dec^−1^. As for HER performance, the catalyst only required an overpotential of about 28 mV to achieve a current density of 10 mA cm^−2^ and a Tafel slope of 36 mV dec^−1^. Regarding OWS, it exhibited an approximate potential of 1.58 V at 10 mA cm^−2^ and demonstrated prolonged stability with almost no loss of activity within 24 h.^[^ ^105^ ^]^ Besides, Tang et al. grew a 2D cobalt carbonate hydroxide nanosheet array onto porous and conductive NF. The authors found that the doping of Mn modulated the morphology and composition of the resultant catalyst at the same time (Figure 18d,e). Due to the abundance of active sites and the optimized electronic structure, this heterostructure displayed excellent OWS activity with a cell voltage of only 1.68 V at 10 mA cm^−2^. Furthermore, the catalyst showed stable electrocatalytic performance with a current density of 1000 mA cm^−2^ at an overpotential of just 462 mV.^[^ ^106^ ^]^

As mentioned earlier, MOFs possess porous, high specific surface, and tailorable features. On the other hand, MOFs also exhibit low mass transmission due to the obstruction of active metal centers. To address this challenge, heterostructures formed by 2D layered materials and MOF structures can effectively enhance catalytic efficiency. For example, Chen et al. successfully prepared a heterostructure consisting of superfine Co NPs embedded in 2D N‐doped carbon NSs and 3D hollow carbon polyhedral through an electrodeposition and pyrolysis method (Figure 18f,g). The braced CC structure inhibited the possible aggregation or self‐stacking of 2D and 3D subunits. In addition, the presence of numerous active sites and fast charge transfer ability contributed to the excellent catalytic properties exhibited by this catalyst. As expected, the catalyst only required overpotentials of 66 and 248 mV to reach 10 mA cm^−2^ for HER and OER, respectively, under alkaline conditions. For OWS catalytic performance, it required a cell potential of 1.545 V to reach a current density of 10 mA cm^−2^ in an alkaline electrolyze and showed good stability.^[^ ^107^ ^]^ Similarly, Zhang et al. utilized Co‐based MOFs to synthesize 3D Co/Co_9_S_8_ core–shell structures anchored onto 2D S, N co‐doped porous graphene sheets (Figure 18h–j). This Co‐MOFs@GO heterostructure possessed a large pore volume and a high active area, which were beneficial to the performance of the catalytic reaction. As a result, the catalyst showed an overpotential of 290 mV for OER at 10 mA cm^−2^ and 350 mV for HER at 20 mA cm^−2^ in a 0.1 m KOH solution.^[^ ^108^ ^]^ Furthermore, 2D materials can also self‐assemble to form 3D structures. For instance, Song et al. prepared 3D architectures via the self‐assembly of 2D nanosheets, in which Co–Ni–S–P compounds were coupled with graphene (Figure 18k–m). This heterostructure had appropriate dual tuning of Ni and P and rich active sites, resulting in excellent catalytic activity. The catalyst showed a cell potential of 1.65 V at 10 mA cm^−2^ for OWS and remained stable after 50 h.^[^ ^109^ ^]^

## 3 CO_2_RR

The excessive emission of CO_2_ is the main problem that can seriously threaten humanity and the environment.^[^ ^5^ ^]^ The electrochemical conversion of CO_2_ to carbon monoxide (CO),^[^ ^172^ ^]^ carboxylic acids,^[^ ^173^, ^174^ ^]^ hydrocarbons,^[^ ^175^ ^]^ and alcohols^[^ ^176^, ^177^ ^]^ can tackle environmental challenges, such as the greenhouse effect caused by carbon dioxide emissions, and provide fuel energy.^[^ ^178^ ^]^ However, the stable thermodynamic and kinetic properties of CO_2_ make the conversion difficult and require a catalyst to reduce the reaction overpotential.^[^ ^179^ ^]^ Heterostructure‐based electrocatalysts have displayed great prospects in CO_2_ conversion due to the synergistic effect among materials, substantial active sites, and high conductivity.^[^ ^180^ ^]^ The final products of CO_2_ reduction depend on the electrocatalyst employed in the electrochemical reaction.^[^ ^181^ ^]^ As known, doping is an efficient strategy to upgrade the electrocatalytic efficiency of CO_2_RR. For instance, Liu et al. used N‐doped carbon metal‐free materials as electrocatalysts for converting CO_2_ into CO. The electrocatalyst showed an onset overpotential of −0.16 V and a CO Faradaic efficiency (FE) of 95%.^[^ ^182^ ^]^

Copper, being a nonprecious metal, has been extensively studied due to its high selectivity and outstanding overall faradaic efficiency for CO_2_RR.^[^ ^183^ ^]^ Zeng et al. studied the influence of particle size and copper valence state on the selectivity and efficiency of carbon monoxide production. They found that cuprous oxide (Cu_2_O) exhibited a higher CO catalytic selectivity compared to cupric oxide (CuO), with the highest selectivity observed when the particle size was the smallest.^[^ ^184^ ^]^ Yoshihara et al. investigated the impact of the surface structure of Cu on catalytic selectivity through etching. The results showed that the catalytic selectivity of hydrocarbons depended on the etchant chosen.^[^ ^185^ ^]^ In comparison with other metallic elements, Cu‐based catalyst materials have several advantages, such as weak adsorption of H and moderate adsorption of CO. The weak adsorption of H allows Cu to inhibit the HER during the electrochemical reaction and improve the Faraday efficiency of carbon‐containing products. Simultaneously, the moderate adsorption of CO ensures a certain level of activity of Cu in CO_2_RR. Due to the distinct binding energies of metal catalysts and intermediates during CO_2_RR and HER, hydrocarbons are the main product of Cu in CO_2_RR.^[^ ^186^ ^]^ Therefore, Cu‐based catalyst materials are extensively used in CO_2_RR. However, as the requirements for catalyst material performance continue to improve, certain aspects of single metal Cu‐based catalytic reactions still need improvement, including slow reaction kinetics, low product selectivity, and poor stability. These issues can be addressed by introducing heterostructures into Cu‐based catalyst materials.

In addition, through the adjustment of the interface between the catalyst and the 2D plane, both the conductivity of the heterostructure and the efficiency of CO_2_ reduction can be improved. For example, Wang et al. discovered that the benzene rings of diphenyl sulfide exhibited a robust face‐to‐face stacking with graphene. By introducing diphenyl sulfide as an axial ligand, they were able to improve the interfacial electron transmission of tetraphenyl porphyrin cobalt (PCo) immobilized onto graphene.^[^ ^188^ ^]^

### 3.1 CO_2_RR on 2D–0D Heterostructures

**Figure 19:**
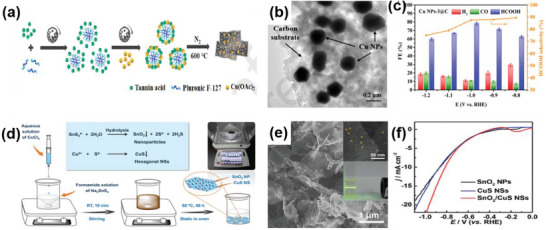
a) Scheme for the formation of Cu NPs@C heterostructure. b) TEM image of Cu NPs‐3@C. c) FE for all products and HCOOH selectivity versus potentials of Cu NPs‐3@C. Reproduced with permission.^[^ ^110^ ^]^ Copyright 2020, Elsevier B.V. d) Schematic illustration for the synthesis of SnO_2_/CuS heterostructure. e) SEM image of SnO_2_/CuS heterostructure. f) LSV curves of in CO_2_‐saturated 0.1 m KHCO_3_ solution. Reproduced with permission.^[^ ^111^ ^]^ Copyright 2020, Royal Society of Chemistry.

Cu NPs can serve as 0D materials to prepare 2D–0D heterostructures with 2D nanosheets. For example, Yang et al. used a ball‐milling method to prepare Cu NPs embedded in a carbon substrate to convert CO_2_ to HCOOH (**Figure** **19**a,b). The fast charge transfer ability promoted the formation of intermediates. As a result, the catalyst exhibited a high FE of 78% for HCOOH at −1.0 V (RHE) (Figure 19c).^[^ ^110^ ^]^ Additionally, 2D planes of Cu‐based materials can also be prepared to form heterostructures. Synthesizing Cu‐based 2D materials can improve the selectivity and efficiency of the CO_2_RR process. As shown in Figure 19d, Wang et al. prepared 0D SnO_2_ NPs confined on ultrathin 2D CuS nanosheets under mild conditions to construct a novel hierarchical 0D/2D heterostructure. It can be observed from Figure 19e that ultrasmall SnO_2_ NPs were grown on thin nanosheets. Compared to SnO_2_ NPs and CuS nanosheets alone, the heterostructure showed the best activity attributed to strong electronic interaction (Figure 19f). Moreover, by modulating the Sn/Cu ratio, the size, and morphology of the catalyst product varied, thereby adjusting the proportion of syngas (CO and H_2_).^[^ ^111^ ^]^

With the wide application of single‐atom (SA) catalysis, Cu SA holds great potential as a 0D material in the 2D–0D heterostructure. The preparation of 2D–0D catalysts consisting of Cu SAs can improve the catalytic performance of CO_2_RR. In this regard, Feng et al. analyzed the catalytic properties of Cu SAs immobilized on B‐N‐doped graphdiyne through first‐principle computations.^[^ ^189^ ^]^

### 3.2 CO_2_RR on 2D–1D Heterostructures

**Figure 20:**
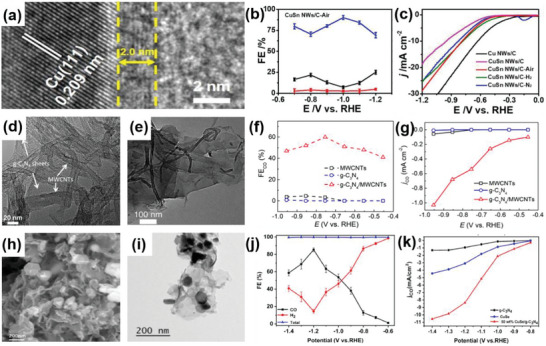
a) HRTEM image of CuSn NWs. b) FE of H_2_, CO, and HCOOH for CuSn NWs/C‐Air. c) LSV curves of CuSn NWs under different conditions. Reproduced with permission.^[^ ^112^ ^]^ Copyright 2019, Elsevier Ltd. d,e) TEM images of the g‐C_3_N_4_/MWCNTs heterostructure. f) The relationship between FE_CO_ and applied potential for CO_2_RR. g) Partial current density of CO of g‐C_3_N_4_/MWCNTs heterostructure and other samples. Reproduced with permission.^[^ ^114^ ^]^ Copyright 2016, Wiley‐VCH. h,i) SEM and TEM images of CuSe/g‐C_3_N_4_ heterostructure. j) FE plots of CuSe/g‐C_3_N_4_ heterostructure. k) partial current density of CO of CuSe/g‐C_3_N_4_ heterostructure and other samples. Reproduced with permission.^[^ ^115^ ^]^ Copyright 2021, Elsevier Ltd.

1D nanowires possess some advantages, including inherent anisotropy and high flexibility. Additionally, by modulating the aspect ratio of 1D nanowires, the electronic properties of the catalyst can be tuned, which has an important influence on the binding strength of the intermediate products during CO_2_RR. For example, Wang et al. prepared CuSn core–shell nanowires deposited on carbon using hydrothermal and annealing methods (**Figure** **20**a–c). When annealed at 300 °C for 1 h in air, the interior metallic Cu gradually diffused outward and transformed into CuO. The performance testing results showed that the catalyst annealed in air exhibited the best selectivity of 90.2% at −1.0 V versus RHE, mainly due to the synergy between Sn atoms on the CuO (111) surface and the modulation of the structure's electronic properties.^[^ ^112^ ^]^ Similarly, Chen et al. coated a two‐phase 2D CuO shell on 1D Cu nanowires, serving as an effective electrocatalyst for CO_2_ conversion to syngas (H_2_/CO). The high‐resolution TEM image confirmed the coating of shells on the copper nanowires, exhibiting different crystal structures and orientations. In particular, the square lattice, representing the face‐centered lattice, exhibited a metastable phase. By adjusting the water‐splitting reaction rate on the electrocatalyst, the ratio of syngas was controlled. Owing to the strong adsorption of intermediate and fast kinetics in CO generation, this heterostructure exhibited a high FE of up to 90%.^[^ ^113^ ^]^

The introduction of g‐C_3_N_4_ is also beneficial for CO_2_RR because the carbon in g‐C_3_N_4_ can exhibit high oxyphilicity, allowing it to adsorb reaction intermediates that bind to oxygen. For instance, Lu et al. utilized a solution reaction to synthesize a 2D–1D g‐C_3_N_4_/MWCNT heterostructure, which served as a stable electrocatalyst for CO_2_RR (Figure 20d–g). The g‐C_3_N_4_/MWCNT composite possessed rich active sites provided by the covalent C─N bonds, a large surface area, and high conductivity, greatly enhancing the electrocatalytic activity and stability of CO_2_RR.^[^ ^114^ ^]^

### 3.3 CO_2_RR on 2D–2D Heterostructures

Due to the advantages of Cu‐based materials in CO_2_RR, 2D Cu‐based materials are the primary consideration for forming 2D–2D heterostructures. For instance, Zhang et al. anchored hexagonal CuSe nanoplates onto g‐C_3_N_4_ nanosheets to prepare a 2D–2D CuSe/g‐C_3_N_4_ heterostructure electrocatalyst for CO_2_RR (Figure 20h–k). The planar structure of g‐C_3_N_4_ provided a large specific area, and the speed of electron transfer was accelerated via the interface between CuSe and g‐C_3_N_4_. As a result, the CuSe/g‐C_3_N_4_ catalyst exhibited a high FE of 85.28%.^[^ ^115^ ^]^

## 4 NRR

The content of N_2_ in the earth accounts for ≈78% of the total air.^[^ ^190^ ^]^ Synthetic ammonia (NH_3_), which is derived from N_2_, occupies a prominent position in human development. Additionally, NH_3_ serves as the raw material for most of the large N‐containing fertilizers in the industry. In addition, NH_3_ is an important inorganic chemical product that is also utilized in the production of other chemical products. However, due to the inert nature of N_2_, the synthesis of NH_3_ using a Haber–Bosch catalyst necessitates high temperature and pressure conditions.^[^ ^191^ ^]^ Consequently, heterostructure catalysis can become a great alternative for improving the efficiency of N_2_ reduction.

### 4.1 NRR on 2D–0D Heterostructures

**Figure 21:**
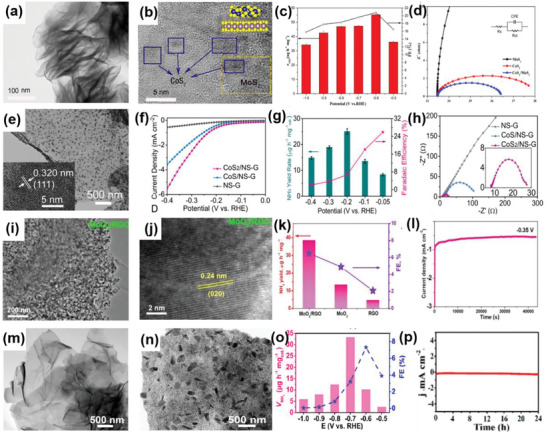
a,b) TEM and HRTEM images of CoS_2_/MoS_2_ heterostructure. c) NH_3_ yields and FEs of CoS_2_/MoS_2_. d) Nyquist plots of CoS_2_/MoS_2_ and other samples. Reproduced with permission.^[^ ^116^ ^]^ Copyright 2021, American Chemical Society. e) TEM image and corresponding HRTEM image (inset) for CoS_2_/NS‐G heterostructure. f,g) Polarization curves and NH_3_ yields and FEs of CoS_2_/NS‐G. h) Nyquist plots of CoS_2_ /NS‐G and other samples. Reproduced with permission.^[^ ^117^ ^]^ Copyright 2019, National Academy of Science. i,j) TEM and HRTEM images of MoO_2_/rGO heterostructure. k) NH_3_ yields and FEs of MoO_2_/rGO. l) Chronoamperometry test of MoO_2_/rGO for 12 h. Reproduced with permission.^[^ ^118^ ^]^ Copyright 2018, Royal Society of Chemistry. m,n) TEM images of rGO and Cr_2_O_3_–rGO heterostructure. o) NH_3_ yields and FEs of Cr_2_O_3_–rGO. p) Chronoamperometry test of Cr_2_O_3_–rGO for 24 h. Reproduced with permission.^[^ ^119^ ^]^ Copyright 2019, American Chemical Society.

Recently, 2D–0D CoS_2_‐based heterostructures have been confirmed as excellent candidates for NRR. For example, Yang et al. prepared a CoS_2_/MoS_2_ heterostructure material as a catalyst for NRR (**Figure** **21**a–d). It can be observed that 0D CoS_2_ NPs were decorated on 2D MoS_2_ nanosheets. The strong interaction between them played a role in transferring electrons from CoS_2_ to MoS_2_. Thus, this transfer helped to form an electrophilic area near CoS_2_, leading to the efficient absorption of N_2_. Besides, the existence of the nucleophilic region near MoS_2_ made it easier to break N≡N. With their combined efforts, this heterostructure exhibited excellent NRR performance, including an NH_3_ yield of 54.7 µg h^−1^ mg^−1^ and FE of 20.8% at −0.6 V, outperforming most MoS_2_‐based electrocatalysts.^[^ ^116^ ^]^ Moreover, Chen et al. used an in situ annealing method to grow 0D CoS_2_ NPs on 2D graphene sheets to form a CoS_2_/NS‐G catalyst (Figure 21e). Because of the presence of Co─N/S─C bridging bonds, this heterostructure possessed a strong binding force between CoS_2_ and graphene, which was beneficial for catalytic performance. As expected, CoS_2_/NS‐G showed high NRR activity with a FE_NH3_ of 25.9% at −50 mV (vs RHE) (Figure 21f–h).^[^ ^117^ ^]^

In addition to 2D–0D CoS_2_‐based heterostructures, Wang et al. prepared 0D transition metal oxide MoO_2_ NPs on 2D graphene for NRR (Figure 21i–l). DFT calculations revealed that the Mo edge sites could absorb the intermediate *N_2_H and were the main active sites for NH_3_ synthesis. Compared with MoO_2_ alone, the formed heterostructure had a much stronger binding force and a more rapid electronic transmission. Moreover, MoO_2_ had inferior HER activity, which was conducive to enhancing FE. As a result, the formation of the heterostructure improved NH_3_ yields and the FE of the catalyst.^[^ ^118^ ^]^ Changing the catalyst material from MoO_2_ to Mo_2_N can further optimize the catalytic activity. For instance, Ren, et al. first synthesized MoO_2_ and then nitrided it to obtain Mo_2_N. The NRR performance was significantly enhanced after conversion, with an NH_3_ yield of 78.4 µg h^−1^ mg^−1^ and a FE of 4.5% at −0.3 V (RHE).^[^ ^192^ ^]^ Benefiting from the advantages of rGO, it can also integrate with other materials to create excellent catalysts for NRR. Xia et al. adopted a hydrothermal reaction and annealing method to prepare a heterostructure electrocatalyst in which 0D Cr_2_O_3_ NPs were uniformly grown on 2D rGO (Figure 21m–p). This heterostructure exhibited outstanding activity and selectivity during NRR. The good dispersion, rich active sites, and enhanced conductivity contribute to Cr_2_O_3_/rGO achieving a high NH_3_ yield of 33.3 µg h^−1^mg^−1^.^[^ ^119^ ^]^ Besides, Du et al. designed a 2D–0D Ni@MXene heterostructure electrocatalyst, in which Ni NPs were loaded onto MXene (V_4_C_3_T*_x_*). During NRR, DFT simulations revealed that the synergistic effect between Ni NPs and the O vacancy of V_4_C_3_T*_x_* made a big difference in improving NRR activity. Thus, the Ni@MXene catalyst achieved a high FE of 14.86% and an NH_3_ yield rate of 21.29 µg h^−1^ mg^−1^ at 0.2 mA cm^−2^.^[^ ^120^ ^]^

### 4.2 NRR on 2D–2D Heterostructures

**Figure 22:**
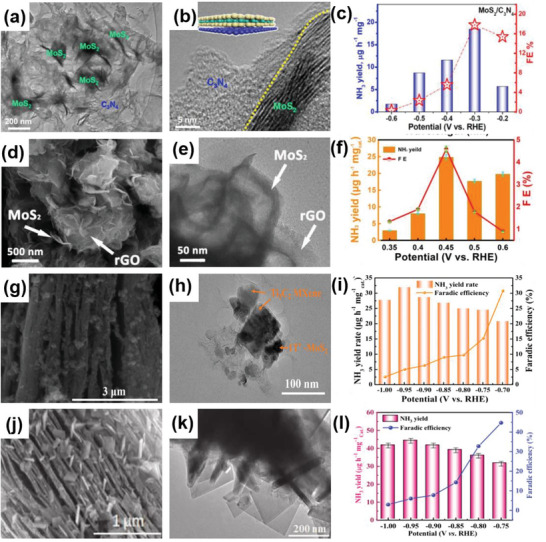
a,b) TEM and HRTEM images of MoS_2_/C_3_N_4_. c) NH_3_ yields and FEs of MoS_2_/C_3_N_4_. Reproduced with permission.^[^ ^121^ ^]^ Copyright 2020, American Chemical Society. d,e) SEM and TEM images of the MoS_2_/rGO heterostructure. f) NH_3_ yields and FEs of MoS_2_/rGO. Reproduced with permission.^[^ ^122^ ^]^ Copyright 2019, Royal Society of Chemistry. g,h) SEM and TEM images of 1T′‐MoS_2_/Ti_3_C_2_ heterostructure. i) NH_3_ yields and FEs of 1T′‐MoS_2_/Ti_3_C_2_. Reproduced with permission.^[^ ^123^ ^]^ Copyright 2022, Elsevier B.V. j,k) SEM and TEM images of TiO_2_/Ti_3_C_2_T*_x_* heterostructure. l) NH_3_ yields and FEs of TiO_2_/Ti_3_C_2_T*_x_* heterostructure. Reproduced with permission.^[^ ^124^ ^]^ Copyright 2022, Elsevier B.V.

The interface effect between 2D materials can improve the free energy of intermediate products and the catalytic efficiency of N_2_ reduction. As shown in **Figure** **22**a–c, Chu et al. synthesized 2D–2D MoS_2_/C_3_N_4_ heterostructures through hydrothermal and liquid exfoliation. Compared with the individual MoS_2_ and C_3_N_4_ components, the efficiency greatly improved. The authors found that the Mo sites had strong electronic interactions with *N_2_H, while S edge sites could adsorb *H, leading to Mo edge sites being less competitive for HER. As a result, the catalyst showed an NH_3_ yield of 18.5 µg h^−1^ mg^−1^ and a high FE of 17.8% at −0.3 V (RHE).^[^ ^121^ ^]^ Due to the advantages of Mo sites, Li et al. synthesized a 2D–2D MoS_2_ nanosheets/rGO nanosheets heterostructure catalyst through thermal treatment (Figure 22d,e). The MoS_2_/rGO heterostructure possessed a high surface area and a strong coupling effect, which were conducive to improving the performance during NRR. As expected, the catalyst showed a FE of 4.58% and an NH_3_ yield of 24.82 µg h^−1^ mg^−1^ at −450 mV (vs RHE) (Figure 22f).^[^ ^122^ ^]^

As an emerging 2D material, MXenes are also potential candidates in the field of catalysis due to their high active surface area and conductivity. For instance, Chen et al. synthesized 2D 1T′‐MoS_2_ nanosheets supported by 2D Ti_3_C_2_ MXene through a hydrothermal method (Figure 22g–i). When the formed 1T′‐MoS_2_/Ti_3_C_2_ heterostructure was used as an NRR electrocatalyst, it achieved an NH_3_ yield rate of 31.96 µg h^−1^ mg^−1^ at −0.95 V and reached a FE of 30.75% at −0.7 V. The catalytic results showed that no by‐product N_2_H_4_ was detected in the electrolyte, indicating great selectivity. Meanwhile, a very slight change in the NH_3_ yield rate and FE after 6 cycles demonstrated its excellent catalytic stability.^[^ ^123^ ^]^ Additionally, Qian et al. synthesized 2D TiO_2_ nanosheets grown in situ on the 2D Ti_3_C_2_T*_x_* nanosheets using a one‐step hydrothermal oxidation process (Figure 22j–l). In terms of structure, the heterostructure facilitated the transfer of charges at the interface and prevented the aggregation of Ti_3_C_2_T*_x_* MXene. Besides, due to the introduction of TiO_2_ nanosheets, the TiO_2_/Ti_3_C_2_T*_x_* heterostructure had a larger surface area than Ti_3_C_2_T*_x_* alone, which was beneficial for enhancing the NRR activity. Furthermore, the rapid electron transfer was inextricably linked to the MXene with high electronic conductivity. Combining the aforementioned factors, the 2D–2D TiO_2_/Ti_3_C_2_T*_x_* heterostructure exhibited good NRR performance with a high NH_3_ yield of 44.17 µg h^−1^ mg^−1^ at −0.95 V and a FE of 44.68% at −0.75 V versus RHE.^[^ ^124^ ^]^

### 4.3 NRR on 2D–3D Heterostructures

**Figure 23:**
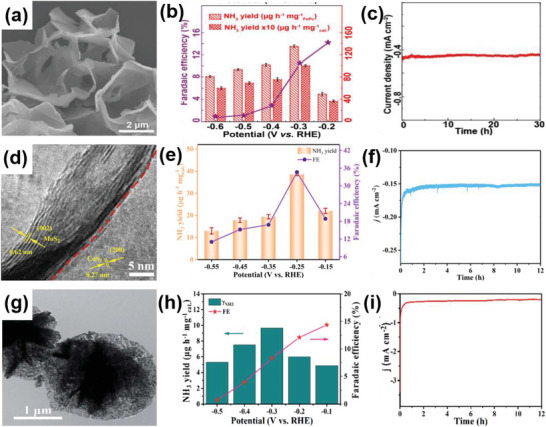
a) SEM image of FePc/C heterostructure. b) NH_3_ yields and FEs of FePc/C heterostructure. c) Chronoamperometry test of FePc/C heterostructure. Reproduced with permission.^[^ ^125^ ^]^ Copyright 2019, American Chemical Society. d) HRTEM image of CoS_2_/MoS_2_ heterostructure. e) NH_3_ yields and FEs of CoS_2_/MoS_2_ heterostructure. f) Chronoamperometry test of CoS_2_/MoS_2_ heterostructure. Reproduced with permission.^[^ ^126^ ^]^ Copyright 2021, Elsevier Inc. g) TEM image of NiS@MoS_2_. h) NH_3_ yields and FEs of NiS@MoS_2_. i) Chronoamperometry test of NiS@MoS_2_. Reproduced with permission.^[^ ^127^ ^]^ Copyright 2021, Royal Society of Chemistry.

2D–3D heterostructures have also been demonstrated to possess good NRR catalytic performance. For example, He et al. synthesized a FePc/C electrocatalyst for NRR by coating iron phthalocyanine (FePc) on pyrolyzing potassium citrate. As displayed in **Figure** **23**a–c, FePc/C maintained a 3D comb‐like structure of interconnected carbon substrates. It was proven that Fe centers were active sites for NRR when compared to Pc/C catalysts without Fe centers. Increasing the exposure of active sites can promote catalytic performance. As a result, FePc/C can generate 10.25 µg h^−1^ mg^−1^ of NH_3_ at a voltage of −0.3 V.^[^ ^125^ ^]^ In addition, Wang et al. adopted a one‐step strategy to prepare a flower‐like 2D–3D CoS_2_/MoS_2_ heterostructure electrocatalyst, which was constituted by dispersed and vertically interconnected nanosheets (Figure 23d–f). The synergistic effect and unique structure with abundant active sites led to excellent catalytic activity, including a high NH_3_ yield rate of 38.61 µg h^−1^ mg^−1^ and a FE of 34.66% at −0.25 V versus RHE.^[^ ^126^ ^]^ Similarly, Huang et al. prepared 2D–3D NiS@MoS_2_ core–shell microspheres formed by MoS_2_ nanosheets on the surface of NiS_2_–NiS (Figure 23g–i). This unique structure provided abundant active sites, high porosity, favorable transport accesses, and other beneficial factors, which were conducive to NH_3_ generation. Consequently, the catalysts showed a high NH_3_ yield rate of 9.66 µg h^−1^ mg^−1^ at −300 mV (vs RHE) and a FE of 14.8% at −100 mV (vs RHE).^[^ ^127^ ^]^

## 5 Conclusion

To conclude, we have systematically reviewed and analyzed the recent studies on 2D materials‐based heterostructures and their applications in the field of energy conversion, including HER, OER, OWS, CO_2_RR, and NRR. We believe that heterostructures are of great help in improving the catalytic activity and catalytic stability of electrocatalytic materials. Considering the abundance of diverse heterostructures, the research and exploration of material structures gradually lead to an understanding of the mechanism. This understanding plays a crucial role in the quest for alternative precious metal catalysts. In the second section, some key parameters in the catalytic reactions of HER, OER, and OWS are introduced. By comparing the electrocatalytic performance of different heterostructures based on key parameters, it is found that: 1) The adjustment of energy level and electron density of the material through the formation of a heterostructure can improve catalytic efficiency; 2) Advantages of different materials, such as high catalytic stability, high catalytic activity, and high conductivity, can be combined. The CO_2_RR is introduced in the third section. It is discovered that Cu exhibits excellent CO_2_RR catalytic efficiency and Faraday efficiency, making Cu‐based materials the main focus of the discussion. In the fourth section, the synthesis method, morphology structure, and unique properties of each heterostructure catalyst for NRR are introduced to understand the reasons for the success of these structures. Additionally, the important challenges during NRR are also addressed, including low reaction selectivity and a comparatively small yield rate.

**Figure 24:**
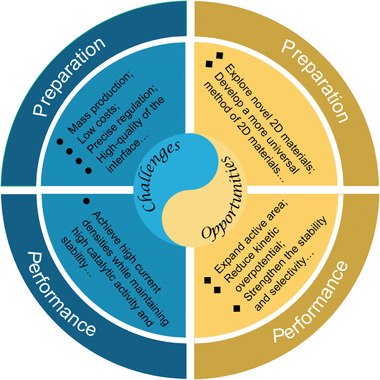
Future challenges and opportunities for 2D materials‐based heterostructures in energy conversion.

Although many remarkable achievements have been produced, there is still room for improvement, and we need to make a huge effort. In this section, we will provide a personal perspective and point out the direction of future in‐depth research (**Figure** **24**).

1. One of the foremost factors limiting the development of 2D materials is the difficulty in their preparation and synthesis. For example, the lack of precursor results in the inability to obtain certain corresponding 2D materials through the peeling process, necessitating alternative approaches for the design and fabrication of these materials. Due to the limited methods available, it is imperative to explore novel and reliable ways to construct heterostructures based on 2D materials. In recent years, researchers have made significant contributions to improving the large‐scale preparation of 2D materials to meet production demands. For example, Zhang et al. developed an intermediate‐assisted grinding exfoliation approach that facilitates the large‐scale production of 2D materials, enabling their commercial applications.^[^ ^193^ ^]^ Similarly, Liu's group reported HER electrocatalysts based on 2D MoS_2_ through a top‐down exfoliation method, allowing mass production while ensuring high catalytic activity.^[^ ^36^ ^]^ Additionally, Choi et al. summarized three recent large‐scale preparation techniques for 2D materials, including CVD, liquid exfoliation, and wet‐chemical synthesis. However, these methods still have limitations and require further development in the future.^[^ ^194^ ^]^ These works provide valuable reference templates for the industrial production of catalysts based on 2D materials and support future research expansion on 2D materials, not limited to just heterostructures. Nonetheless, several main challenges persist in the mass production of catalysts based on 2D materials: i) Purity: Many 2D materials, such as graphene, TMDs, and black phosphorus, are extremely sensitive to impurities, which can significantly impact their properties, including catalytic activity. Therefore, developing purification strategies on an industrial scale is essential to ensure the quality and purity of the final material. ii) Control of the synthesis: The synthesis of 2D materials and heterostructures can be complex due to the dependence of their properties on various parameters such as temperature, pressure, precursor composition, and growth rate. Optimizing these parameters for large‐scale synthesis requires precise control and tight tolerances. iii) Stability: The synthesis of 2D materials and heterostructures demands specific conditions, such as high vacuum or inert environments, to maintain their structural stability. Developing practical methods for handling and preserving these materials during storage and transportation is crucial to ensure their availability and utility in the industry. IV) High‐quality substrates: The growth of 2D materials and heterostructures necessitates high‐quality substrates, which can be expensive and challenging to manufacture consistently, especially on a large scale. Addressing these challenges still requires comprehensive research and continuous efforts in the future.
2. Performance depends on morphology and structure to a certain extent. Designing an appropriate heterostructure based on 2D materials, in combination with different application scenarios, can further improve catalytic efficiency. Additionally, the quality of the interface (such as the number of defects, the electronic structure, etc.) also affects catalytic activity.
3. In the process of reviewing this state‐of‐the‐art research, it can be noticed that the formation of heterostructures is predominantly centered around classical 2D materials, including TMCs, TMDs, graphene, MXene, LDHs, etc. If innovative 2D materials can be designed and created in the future, the actual application performance of heterostructures based on 2D materials will enter a new era. Furthermore, concerning practical applications, some performance issues still exist, such as relatively high overpotential or low current density. The problem of low current density in hydrogen and oxygen evolution has also received attention and improvements in recent years. For example, Luo et al. prepared cost‐effective electrocatalysts, including flat Pt foil and MoS_2_ microspheres, which still exhibit good HER performance at a high current density of 1000 mA cm^−2^.^[^ ^59^ ^]^ Additionally, Liu et al. constructed a CuMo_6_S_8_/Cu electrocatalyst for HER, which can maintain stable and effective operation for over 100 h under a high current density of up to 2500 mA cm^−2^.^[^ ^195^ ^]^ One of the main challenges in hydrogen and oxygen evolution reactions is to achieve high current densities while maintaining high catalytic activity and stability. Several factors limit the maximum current density that can be achieved, including i) Mass transport limitations: The rate of electron transfer at the electrode interface depends on the availability of reactants and products. In the case of hydrogen and oxygen evolution, the mass transport of hydrogen ions, water, and oxygen in the electrolyte can restrict the reaction rate, leading to lower current densities. ii) Catalyst stability: High current densities for extended periods can cause catalysts degradation, resulting in decreased efficiencies and reduced lifetimes. Maintaining the stability of the catalysts under high current densities is critical for achieving efficient and prolonged operation. iii) Catalyst design: The design of the catalysts, including the choice of materials, morphology, and surface area, can impact catalytic activity and stability. Developing catalysts optimized for high current densities requires an in‐depth understanding of the electrochemical mechanisms and material properties. To meet the demand for high current density in industrial production, more attention needs to be given to these challenges. Further research and progress are still required in this area in the future. Improving 2D materials‐based heterostructures in this regard will greatly enhance their potential in industrial applications.
4. Despite much progress made in improving important performance indicators, they still remain a bit far from the ideal goal. Therefore, the uphill struggle we will face in the development of electrocatalysts is how to further expand their active area, reduce kinetic overpotential, and enhance their stability and selectivity.

## Conflict of Interest

The authors declare no conflict of interest.
